# The Micronemal *Plasmodium* Proteins P36 and P52 Act in Concert to Establish the Replication-Permissive Compartment Within Infected Hepatocytes

**DOI:** 10.3389/fcimb.2018.00413

**Published:** 2018-11-27

**Authors:** Silvia A. Arredondo, Kristian E. Swearingen, Thomas Martinson, Ryan Steel, Dorender A. Dankwa, Anke Harupa, Nelly Camargo, William Betz, Vladimir Vigdorovich, Brian G. Oliver, Niwat Kangwanrangsan, Tomoko Ishino, Noah Sather, Sebastian Mikolajczak, Ashley M. Vaughan, Motomi Torii, Robert L. Moritz, Stefan H. I. Kappe

**Affiliations:** ^1^Center for Global Infectious Disease Research, Seattle Children's Research Institute, Seattle, WA, United States; ^2^Institute for Systems Biology, Seattle, WA, United States; ^3^Department of Pathobiology, Faculty of Science, Mahidol University, Bangkok, Thailand; ^4^Department of Molecular Parasitology, Proteo-Science Center, Ehime University, Shitsukawa, Toon, Japan

**Keywords:** malaria, *Plasmodium*, protein complex, invasion, sporozoite, 6-cys s48/45, microneme secretion, TRAP

## Abstract

Within the liver, *Plasmodium* sporozoites traverse cells searching for a “suitable” hepatocyte, invading these cells through a process that results in the formation of a parasitophorous vacuole (PV), within which the parasite undergoes intracellular replication as a liver stage. It was previously established that two members of the *Plasmodium* s48/45 protein family, P36 and P52, are essential for productive invasion of host hepatocytes by sporozoites as their simultaneous deletion results in growth-arrested parasites that lack a PV. Recent studies point toward a pathway of entry possibly involving the interaction of P36 with hepatocyte receptors EphA2, CD81, and SR-B1. However, the relationship between P36 and P52 during sporozoite invasion remains unknown. Here we show that parasites with a single *P52* or *P36* gene deletion each lack a PV after hepatocyte invasion, thereby pheno-copying the lack of a PV observed for the *P52*/*P36* dual gene deletion parasite line. This indicates that both proteins are equally important in the establishment of a PV and act in the same pathway. We created a *Plasmodium yoelii* P36^mCherry^ tagged parasite line that allowed us to visualize the subcellular localization of P36 and found that it partially co-localizes with P52 in the sporozoite secretory microneme organelles. Furthermore, through co-immunoprecipitation studies *in vivo*, we determined that P36 and P52 form a protein complex in sporozoites, indicating a concerted function for both proteins within the PV formation pathway. However, upon sporozoite stimulation, only P36 was released as a secreted protein while P52 was not. Our results support a model in which the putatively glycosylphosphatidylinositol (GPI)-anchored P52 may serve as a scaffold to facilitate the interaction of secreted P36 with the host cell during sporozoite invasion of hepatocytes.

## Introduction

Malaria-causing *Plasmodium* parasites are responsible for taking nearly half a million lives worldwide every year (WHO, 2017). *Plasmodium* transmission occurs when sporozoites are deposited in the skin by a feeding, infected *Anopheles* mosquito. By means of gliding motility and cell traversal, sporozoites cross skin tissue and enter blood capillaries which allow their transport to the liver where they invade hepatocytes and form liver stages. Following parasite growth and replication within a hepatocyte, tens of thousands of merozoites will be released into the blood stream initiating the asexual blood cycle, leading to symptomatic malaria disease and possibly death. Before successfully establishing a liver infection, sporozoites will traverse several cell types, including hepatocytes within transient vacuoles (TV), searching for a suitable host hepatocyte. Upon encountering such a cell, sporozoites switch to “invasion mode” and enter by creating the replication-permissive parasitophorous vacuole (PV) (Mota et al., 2001; Risco-Castillo et al., 2015). The PV is vital for the survival and normal progression of liver stage development as it separates the parasite from the host cell cytoplasm with a host-derived membrane remodeled by the parasite (the PV membrane, PVM) (Lingelbach and Joiner, 1998; Nyboer et al., 2017). The conserved *Plasmodium* proteins P36 and P52 have been linked to the establishment and/or maintenance of the PV following the observation that intracellular Δ*P52*Δ*P36* dual gene deletion parasites do not display a PVM as analyzed by electron microscopy a few hours after infection (Labaied et al., 2007; Ploemen et al., 2012). However, the respective contributions of P52 and P36, their molecular interactions, and the mechanisms by which these proteins are involved in invasion remain unknown.

P36 and P52, having two s48/45 structural domains each, belong to the conserved *Plasmodium* 6-cys s48/45 family comprising proteins with crucial functions in parasite fertilization and immune evasion (Gerloff et al., 2005; Arredondo and Kappe, 2016). P36 and P52 are arranged in tandem in the genome and while both have a secretory signal sequence, only P52 is predicted to be GPI-anchored (Templeton and Kaslow, 1999; Thompson et al., 2001). Transcriptional and proteomic studies in *Plasmodium falciparum, berghei*, and *yoelii* indicate that P36 and P52 are expressed in sporozoites (Kappe et al., 2001; Le Roch et al., 2003; van Dijk et al., 2005, 2010; Labaied et al., 2007; Lasonder et al., 2008; VanBuskirk et al., 2009; Lindner et al., 2013), which has also been confirmed by western blot analysis (Ishino et al., 2005) and it is assumed that these proteins are secreted by sporozoites in order to interact with host cells. Upon invasion, the sporozoite begins the sequential release of the apical organelles starting with the micronemes, believed to store proteins that are thought to be important for mediating early events in hepatocyte invasion and establishment of the PVM (Lingelbach and Joiner, 1998; Soldati et al., 2001). Previous work suggested that P52 is localized in the micronemes as shown by immunofluorescence (IFA) and low-resolution immune electron microscopy (EM) (Ishino et al., 2005; VanBuskirk et al., 2009); conversely, no subcellular localization has been reported for P36 thus far.

Previous work showed the essentiality of P36 and P52 for the productive invasion of hepatocytes by sporozoites in rodent malaria models as well as *P. falciparum* (VanBuskirk et al., 2009), including clinical data with a Δ*P52*Δ*P36* dual gene deletion genetically attenuated parasite (GAP) strain (Spring et al., 2013). Deletion of either gene alone or both genes has no effect on parasite blood stage proliferation, development within the mosquito or colonization of salivary glands, and *in vitro* and *in vivo* studies show that Δ*P36*, Δ*P52*, and Δ*P52*Δ*P36* dual gene deletion sporozoites are able to enter and traverse hepatocytes equally well as wild-type parasites. However, these mutants stop developing soon after invasion, and are promptly cleared from mouse livers possibly through apoptosis of the infected cell (van Dijk et al., 2005; Kaushansky et al., 2015), resulting in greatly reduced host infection (Ishino et al., 2005; van Dijk et al., 2005; Douradinha et al., 2007; Labaied et al., 2007; van Schaijk et al., 2008; VanBuskirk et al., 2009; Annoura et al., 2012; Ploemen et al., 2012). Simultaneous deletion of *P36* and *P52* reportedly leads to a somewhat stronger attenuation phenotype in comparison to single deletion mutants (Labaied et al., 2007; VanBuskirk et al., 2009).

While both P36 and P52 are important for sporozoite invasion, it remained unknown how these proteins interact with the hepatocyte surface and only P36 has been recently implicated to directly mediate host cell interactions. First, a functional connection of P36 with EphA2, a host cell receptor involved in hepatocyte susceptibility to infection and formation of the PVM, was proposed following the observation that signaling of EphA2 upon interaction with its natural ligand EphrinA1 was disrupted in the presence of P36 (Kaushansky et al., 2015). Thereafter, a series of cross-*Plasmodium* species complementation experiments reported that while P52 plays a conserved role in sporozoite invasion in all examined *Plasmodium* species, the choice of route by which parasites invade hepatocytes is determined by P36 in a species-specific manner, establishing a functional link for P36 with the host receptors CD81 and SR-BI, the former also known to be required for PVM formation (Manzoni et al., 2017). However, a direct physical interaction of P36 with the host cell receptors was not demonstrated in any of these studies.

Given the critical roles of P36 and P52 during hepatocyte invasion, it is essential to gain insights into how these proteins act, whether they act in the same pathway and whether they exert their function together or individually. Here, we show that each protein is necessary for the formation of the PVM, determine unequivocally the localization of P36 and P52 and their co-localization within the sporozoite micronemes, establish that P36 is secreted upon sporozoite stimulation but P52 is not, and demonstrate that P36 forms a complex with P52. We conclude that P36 and P52 act as a complex within the same sporozoite infection pathway leading to host cell engagement and subsequent induction of PVM formation, which is a prerequisite for productive infection of hepatocytes.

## Materials and methods

### Creation of *P. yoelii* RFP-tagged lines

The Δ*P36*, Δ*P52* (PlasmoDB identifiers PY17X_1003500 and PY17X_1003600, respectively), and Δ*P52*Δ*P36* knockout parasite lines were created by gene replacement through double crossover homologous recombination (Figure S1A) as previously described (Mikolajczak et al., 2008), where a red fluorescent protein (RFP) cassette was inserted in place of each gene, or both genes simultaneously; RFP is expressed in the parasite cytoplasm (oligonucleotides used in this study are listed in Table S1). DsRed fluorescent PyS1^−^ parasites were used as wild-type controls (Jacobs-Lorena et al., 2010).

### Creation of *P. yoelii* P36^mcherry^

The creation of P36^mCherry^ (PlasmoDB identifier PY17X_1003500) relied upon double crossover homologous recombination using a modified plasmid pDEF (van Dijk et al., 2005), which allowed for the addition of a fluorescent mCherry epitope tag to the carboxy terminus of P36 (Figure S1B). The resultant *P. yoelii* P36^mCherry^ parasite expresses a single copy of P36, with a C-terminal mCherry tag, under the control of its endogenous promoter. Briefly, the two regions of the targeted locus were PCR amplified with locus-specific primers (Table S1). The two PCR products were then fused by Sequence Overlap Extension PCR (“SOE PCR”). This PCR product was then digested at the 5′ and 3′ ends with restriction sites incorporated into the primers and inserted into the modified pDEF vector. *P. yoelii* 17XNL parasites were genetically modified using standard methods as previously described (Jongco et al., 2006). The presence of transgenic parasites was assessed by genotyping PCR (Vaughan et al., 2009) and limiting dilution infection of female Swiss Webster mice was used to isolate two independent clones of the transgenic parasite for further analysis. Parasites were cycled through all stages of development and time to patency, as compared to wild-type parasites, was determined by injecting 5 × 10^4^ sporozoites i.v. into Swiss Webster mice and analyzing blood smears for parasites.

### P36^mcherry^ expression

Freshly extracted live sporozoites were analyzed by fluorescence microscopy to visually confirm the expression of P36^mCherry^. Relative expression of P36^mCherry^ and P52 in midgut and salivary gland sporozoites was analyzed by western blot. Briefly, 1 × 10^6^ sporozoites from midguts dissected on day 10 and an equal number of sporozoites from salivary glands dissected on day 14 from the same batch of infected mosquitoes were extracted and pelleted at 6 krpm for 6 min, followed by the creation of lysates by resuspending the sporozoite pellets in 2 × SDS buffer and boiling. Equivalent numbers of uninfected midguts and salivary glands were processed in parallel to use as controls. The lysates and controls were subjected to SDS-PAGE using a 4–12% gradient Bis-Tris gel (Thermo Fisher Scientific), transferred onto a PVDF membrane, blocked for 1 h at room temperature in 5% milk in TBST (Tris buffered saline, 0.1% Tween-20), incubated overnight with α-mCherry (16D7) rat mAb in 2% milk/TBST at 4°C, washed, and incubated with donkey α-rat horseradish peroxidase (HRP)-conjugated IgG in 2% milk/TBST for 1 h at room temperature. The membrane was stripped with Restore (Thermo Fisher Scientific) twice and re-incubated with α-PyP52 (13G10) mouse mAb (Supplementary Methods) and goat α-mouse HRP-conjugated IgG, and α-MTIP rabbit serum and goat α-rabbit HRP-conjugated, respectively. Densitometry analysis was done using ImageJ (Schneider et al., 2012).

### Immunofluorescence assays (IFA)

For *in vitro* invasion assays: Hepa 1–6 mouse cells maintained in complete DMEM media (Thermo Fisher Scientific) containing 10% FBS (Sigma-Aldrich), 200 U/mL penicillin with 200 μg/mL streptomycin (Thermo Fisher Scientific), and 2.5 μg/mL Fungizone (GE Life Sciences) were plated in 8-well chamber slides (1 × 10^5^ cells per well) allowing them to rest overnight. Sporozoites were extracted, activated by incubating with 20% FBS in DMEM for 15 min at room temperature, diluted in complete DMEM at 37°C, and added to the Hepa 1–6 cells to a final concentration of 1 × 10^5^ sporozoites per well in 150 μl total volume. Chamber slides were centrifuged at 500 g for 3 min and invasion was allowed for 90 min for P36^mCherry^ sporozoites or 2 h for RFP-labeled strains at 37°C followed by one quick wash with 1 × PBS. Samples were then fixed with 10% neutral buffered formalin (VWR) for 20 min at room temperature and washed twice with PBS. The slides were kept in a wet chamber at room temperature. Blocking and permeabilization was done simultaneously with 2% (wt/vol) BSA (Calbiochem), 0.2% (vol/vol) Triton X-100 (Thermo Fisher Scientific) in PBS for 3 h. After washing, samples were incubated with primary antibody (Table S4) diluted in blocking solution (2% BSA, in PBS) for 2 h. The sporozoites were then incubated with secondary antibodies (Table S4) in blocking solution for 1 h and washed. Nucleic acid was stained with DAPI (4′,6′-diamidino-2-phenylindole) (Thermo Fisher Scientific) at 1 μg/ml in PBS for 5 min. After washing, slides were mounted with Vectashield HardSet (Vector Laboratories). For filipin labeling: invasion was allowed to proceed for 2 h as described, followed by one wash with PBS, live staining with 25 μg/ml filipin (Sigma) for 10 min, and a final PBS wash prior to analysis.

For sporozoite IFAs: sporozoites (3 μl) were spotted onto a glass slide and allowed to dry overnight; fixing, blocking, permeabilization, staining, and mounting were performed as described above. All slides were analyzed by fluorescence microscopy acquiring images with Olympus 1 × 71 DeltaVision Elite deconvolution microscopy. Imaris software was used for 3D image visualization.

### Immunoelectron microscopy assays (IEM)

Mosquito salivary glands infected with *Py* P36^mCherry^ sporozoites were dissected, collected in cold PBS, and fixed in 1% paraformaldehyde (PFA)/0.2% glutaraldehyde (Electron Microscopy Sciences) in PBS for 20 min on ice with frequent agitation. The glands were washed with PBS, stored in 4% sucrose in PBS at 4°C and then embedded in LR White resin (Polysciences). Sections (70 nm thickness) were blocked for 30 min in blocking buffer (5% non-fat dry milk, 0.01% Tween-20 in PBS), incubated overnight at 4°C in blocking buffer containing mouse and/or rabbit primary antibodies (Table S4), and then incubated for 1 h in blocking buffer containing goat α-mouse IgG conjugated with 15-nm gold particles (British BioCell International) and/or goat α-rabbit IgG conjugated with 10-nm gold particles (Invitrogen, Thermo Fisher Scientific). Sections were stained with 2% uranyl acetate and lead citrate, and examined with a JEM-1230 electron microscope (JEOL, Japan).

### Immunoprecipitation assays

Sporozoites were pelleted by centrifuging at 4 krpm for 4 min, washed once in Schneider's media (Gibco), spun at 6 krpm for 6 min and stored at −80°C. To create lysates, a frozen sporozoite pellet (3–4 × 10^6^ spz) was thoroughly resuspended in 200 μl of ice-cold lysis buffer [10 mM Tris-HCl pH 7.5; 150 mM NaCl; 0.5 mM EDTA; 0.5% NP-40 (Fluka)] containing 2 × protease inhibitor cocktail (Cell Signaling), placed on ice, and vortexed every 10 min for 1 h. Sporozoite lysates were centrifuged at 13 krpm for 20 min at 4°C and the supernatants were collected and mixed with 200 ul of dilution buffer (10 mM Tris-HCl pH 7.5; 150 mM NaCl; 0.5 mM EDTA). P36^mCherry^ lysate, and wild-type lysate as a control for non-specific binding, were incubated with RFP-Trap®_MA beads (Chromotek) to immunoprecipitate mCherry following the manufacturer's instructions with modifications. Briefly, the sporozoite lysates were pre-cleared by incubating with 50 μl of unconjugated control beads at 4°C for 1 h with rotation. After magnetically separating the beads, the lysates were transferred to a new tube containing 50 μl of previously washed RFP-Trap®_MA beads and incubated at 4°C for 1 h with rotation. Control and RFP-Trap®_MA beads were washed four times with dilution buffer, magnetized, and stored at −80°C. Frozen bead pellets were resuspended in 2 × SDS buffer, boiled and magnetized, subjecting supernatants to SDS-PAGE and western blot analysis following the procedure described above; mass spectrometry analysis was also performed in parallel. For the crosslinking experiment, freshly extracted sporozoites were purified on an Accudenz (Accurate Chemical & Scientific Corporation) discontinuous gradient as previously described (Kennedy et al., 2012) and the lysate was created as described using modified lysis and dilution buffers by substituting Tris-HCl with PBS. Following centrifugation of sporozoite lysate, the supernatant was collected, mixed with an equal volume of ice-cold dilution buffer containing 5 mM BS3 (Thermo Fisher Scientific) and incubated on ice for 2 h. The reaction was quenched by adding ammonium bicarbonate (ABC) to a 100 mM final concentration and incubating at room temperature for 15 min. Lysates were analyzed by SDS-PAGE, western blot and mass spectrometry as described.

### Liquid chromatography-mass spectrometry

With the exception of the cross-linked sample, proteins isolated from the RFP-Trap® IP were subjected to SDS-PAGE pre-fractionation and in-gel tryptic digestion essentially as described in Lindner et al. (2013). Briefly, samples were electrophoresed through a 4–12% w/v gradient Bis-Tris gel (Thermo Fisher Scientific). Gels were stained with SimplyBlue Safestain (Thermo Fisher Scientific), destained in Milli Q Water (Millipore), and each lane was cut into four fractions, each of which was cut into small pieces (~1 mm^2^). The following steps were performed on a thermomixer at 800 RPM and 37°C. Gel pieces were de-stained with 50 mM ABC in 50% acetonitrile (ACN) and dehydrated with ACN. Gel pieces were incubated for 30 min in 10 mM dithiothreitol in 100 mM ABC and 20 min in 50 mM iodoacetamide in 100 mM ABC, washed with 50 mM ABC in 50% ACN, dehydrated with 100% ACN, and rehydrated with 6.25 ng/μL sequencing grade trypsin (Promega) in 50 mM ABC. After incubating overnight at 37°C, the supernatant was recovered, and peptides were extracted by incubating the gel pieces 20 min each with 2% v/v ACN and 1% v/v formic acid (FA) in water, then 60% v/v ACN and 1% v/v FA in water, then 100% ACN. The extractions were combined with the digest supernatant, evaporated to dryness in a rotary vacuum, and reconstituted in liquid chromatrography (LC) loading buffer consisting of 2% v/v ACN and 0.2% v/v trifluoroacetic acid (TFA).

The BS3-crosslinked sample was immunoprecipitated with RFP-Trap® and eluted from beads by adding 50 μL of 5%SDS/50 mM Tris pH 8.5 to which 0.5 mM Bond-Breaker TCEP solution (Thermo Fisher Scientific) was added to 19 mM per 100 μL of beads and incubating 10 min at 95°C. A total of 300 μL eluate was collected, to which 8 μL of 1 M iodoacetamide was added and incubated at room temperature in darkness for 20 min. The eluted protein was digested using an S-Trap Micro (Protifi) following the manufacturer's protocol. Briefly, 30 μL of 12% v/v phosphoric acid was added to the eluate, followed by 1.980 mL S-Trap buffer (90% v/v methanol/ 50 mM Tris pH 8.5). The sample was loaded onto the S-trap and washed with S-Trap buffer by centrifugation. Sixty microliters of 50 mM Tris pH 8.5 containing 0.6 μg of trypsin (Promega sequencing grade) was added and the trap was incubated 3 h at 47°C. Peptides were eluted with 40 μL each of 50 mM Tris pH 8.5, 0.2% v/v formic acid, and 50% v/v ACN/0.2% v/v formic acid, dried down in a speed vac, and reconstituted in 20 μL 5% ACN/0.5% TFA.

Except for the crosslinked sample, LC was performed using an Agilent 1100 nano pump with electronically controlled split flow. Peptides were separated on a column with an integrated fritted tip [360 μm outer diameter (O.D.), 75 μm inner diameter (I.D.), 15 μm I.D. tip; New Objective] packed in-house with a 20 cm bed of C18 (Dr. Maisch ReproSil Pur C18 AQ, 120 Å, 3 μm). Prior to each run, sample was loaded onto a trap column consisting of a fritted capillary (360 μm O.D., 150 μm I.D.) packed with a 1 cm bed of the same stationary phase and washed with loading buffer. The trap was then placed in-line with the separation column for the separation gradient. The LC mobile phases consisted of buffer A (0.1% v/v FA in water) and buffer B (0.1% v/v FA in ACN). The separation gradient was as follows: 5–35% B over 60 min, 35–80% B over 10 min, 10 min at 80% B, 80–5% B over 1 min, then 29 min at 5% B. Tandem MS (MS/MS) was performed with an LTQ Velos Pro-Orbitrap Elite (Thermo Fisher Scientific). A top-20 data-dependent acquisition method was employed to select the top precursors for collision-induced dissociation (CID) and analysis in the ion trap. Dynamic exclusion was enabled to exclude a precursor mass with a ± 5 ppm mass tolerance for 30 s after observing it once. Precursor charge state selection was employed to selecting only doubly- and triply-charged precursors. Two nanoLC MS technical replicates were performed for each fraction, with roughly half the available sample injected for each replicate.

The BS3-crosslinked peptides were analyzed using an EASY nLC coupled to a Q-Exactive HF (Thermo Fisher Scientific). Peptides were separated on a column with an integrated fritted tip [360 μm outer diameter (O.D.), 75 μm inner diameter (I.D.), 15 μm I.D. tip; New Objective] packed in-house with a 50 cm bed of C18 (Dr. Maisch ReproSil Pur C18 AQ, 120 Å, 1.9 μm). Prior to each run, sample was loaded onto a trap column (Acclaim PepMap 100, 75 μm I.D. with a 2 cm bed of C18, 3 μm 100 Å) and washed with buffer A. The separation gradient was as follows: 5–35% B over 120 min, 35–80% B over 1 min, 15 min at 80% B, 80% B to 2% B over 1 min, then 20 min at 2% B. A top-15 data-dependent acquisition method was employed to select the top precursors for higher-energy collision-induced dissociation (HCD). Twenty seconds of dynamic exclusion was enabled. Precursor charge state selection was employed to exclude singly-charged precursors and those with charge state 6 and higher. Half the available sample was injected.

### Peak list generation

Mass spectrometer output files were converted to mzML format using msConvert version 3.0.11579 (Kessner et al., 2008) and searched with Comet version 2017.01 rev.1 (Eng et al., 2013). The spectra were searched against a database comprising *P. yoelii* 17 × (Otto et al., 2014) (PlasmoDB v.35, www.plasmodb.org; Aurrecoechea et al., 2009), *Anopheles stephensi* Indian AsteI2.3 (Jiang et al., 2014) (VectorBase, www.vectorbase.org; Jiang et al., 2014), mCherry fluorescent protein (UniProt accession number X5DSL3), the LC calibration standard peptide [Glu-1] fibrinopeptide B, and the common Repository of Adventitious Proteins (v.2012.01.01, The Global Proteome Machine, www.thegpm.org/cRAP). Decoy proteins with the residues between tryptic residues randomly shuffled were generated using a tool included in the Trans-Proteomic Pipeline (TPP) and interleaved among the real entries. The precursor mass tolerance was set to ±10 ppm. Fragment ion bin and offset were set to 1.0005 and 0.4, respectively, for ion trap spectra and 0.02 and 0.0, respectively, for HCD spectra. Semi-tryptic peptides and up to 2 missed cleavages were allowed. The search parameters included a static modification of + 57.021464 Da at Cys for carbamidomethylation by iodoacetamide and potential modifications of +15.994915 Da at Met for oxidation, + 17.026549 Da at peptide N–terminal Gln for deamidation from formation of pyro–Glu, + 18.010565 Da at peptide N–terminal Glu for loss of water from formation of pyro–Glu, + 17.026549 Da at peptide N–terminal carbamidomethylated Cys for deamidation from formation of S–carbamoylmethylcysteine, and + 42.010565 at the N–terminus of the protein (either at N–terminal Met or the N-terminal residue after cleavage of N-terminal Met) for acetylation. An additional variable modification of + 156.0786 at Lys and peptide N-terminus for dead-end BS3. The MS/MS data were analyzed using the TPP (Deutsch et al., 2015) version 5.1.0 Syzygy. Peptide spectrum matches (PSMs) were assigned scores in PeptideProphet (Keller et al., 2002) followed by iProphet (Shteynberg et al., 2011) with the number of sibling peptides (NSP) model disabled. Protein inferences were assigned with ProteinProphet (Nesvizhskii et al., 2003). Only PSMs with iProphet probabilities corresponding to a false discovery rate (FDR) < 1.0% (as determined from the iProphet mixture models) were used for protein quantification by spectal counting. PSMs from degenerate peptides (peptides whose sequences were found in multiple identified proteins) were split among proteins containing that peptide in a weighted fashion (Zhang et al., 2010; Fermin et al., 2011). The relative abundance of proteins in test and control samples was estimated based on the ratio of spectral counts, which were taken as the total number of PSMs identifying peptides from a given protein.

### Secretion assays

Isolated sporozoites (1.6 × 10^6^) were kept on ice, washed once with ice-cold PBS, and split into two equal aliquots. After centrifuging the sporozoites for 4 min at 4 krpm, the pellets were resuspended in 30 ul of ice-cold PBS, incubating the control aliquot on ice and the activated aliquot in a 37°C water bath for 40 min. The aliquots were then centrifuged at 6 krpm for 6 min at 4°C, collecting the supernatants; pellets and supernatants were stored at −80°C. Supernatants were mixed with 4 × SDS buffer, and pellets were fully resuspended in 2 × SDS buffer to create lysates, followed by SDS-PAGE and western blot analysis as previously described.

### Ethics statement

Female Swiss Webster, BALB/cJ and BALB/cByJ mice of 7–8 months of age were utilized in this study as described. Mice were housed in the Center for Infectious Disease Research animal facility, which has an active PHS Animal Welfare Assurance on file with OLAW (A3640-01) and is an AAALAC accredited facility. The facilities and programs of the vivarium are operated in compliance with state law, federal statute and NIH policy. All protocols involving research animals were reviewed and approved by the Center for Infectious Disease Research IACUC (Protocol's#: SK-01, SK-04 and SK-13). Ketamine/Xylazine was used as an anesthetic. Euthanasia methods followed the recommendations of the American Veterinary Medical Association's Panel on Euthanasia. Mice were euthanized by CO_2_ asphyxiation followed by exanguination, performed by qualified and trained personnel.

## Results

### P36 and P52 are each required for the formation of the parasitophorous vacuole

*Plasmodium yoelii* parasites were genetically modified by double crossover recombination to create Δ*P36* and Δ*P52* single gene deletion mutants and a Δ*P52*Δ*P36* dual gene deletion mutant, all expressing red fluorescent protein (RFP) in their cytoplasm (Figure S1). In agreement with previously reported single and double *P36* and *P52* mutants in *P. berghei, P. falciparum*, and *P. yoelii* (Ishino et al., 2005; van Dijk et al., 2005; Labaied et al., 2007; van Schaijk et al., 2008; VanBuskirk et al., 2009; Annoura et al., 2012), these knockout parasite lines developed normally through the blood cycle and within the mosquito vector, displaying sporozoite gliding motility and cell traversal activity comparable to wild-type controls. The ability of these knockout parasites to establish a successful liver infection progressing into an asexual blood cycle in the host (patency) was analyzed by the intravenous (i.v.) injection of sporozoites into BALB/cJ and BALB/cByJ mice (Table S2). While all mice injected with wild-type sporozoites became blood stage patent as expected, none of the 17 mice injected with Δ*P52* sporozoites became patent, and only 2 in 17 mice injected with the Δ*P36* line became patent, the latter exhibited a patency delay of 1 day. In addition, only 2 in 45 mice injected with ΔP*52*ΔP*36* parasites showed a blood infection, and patency in these animals was further delayed by an extra day.

**Figure 1:**
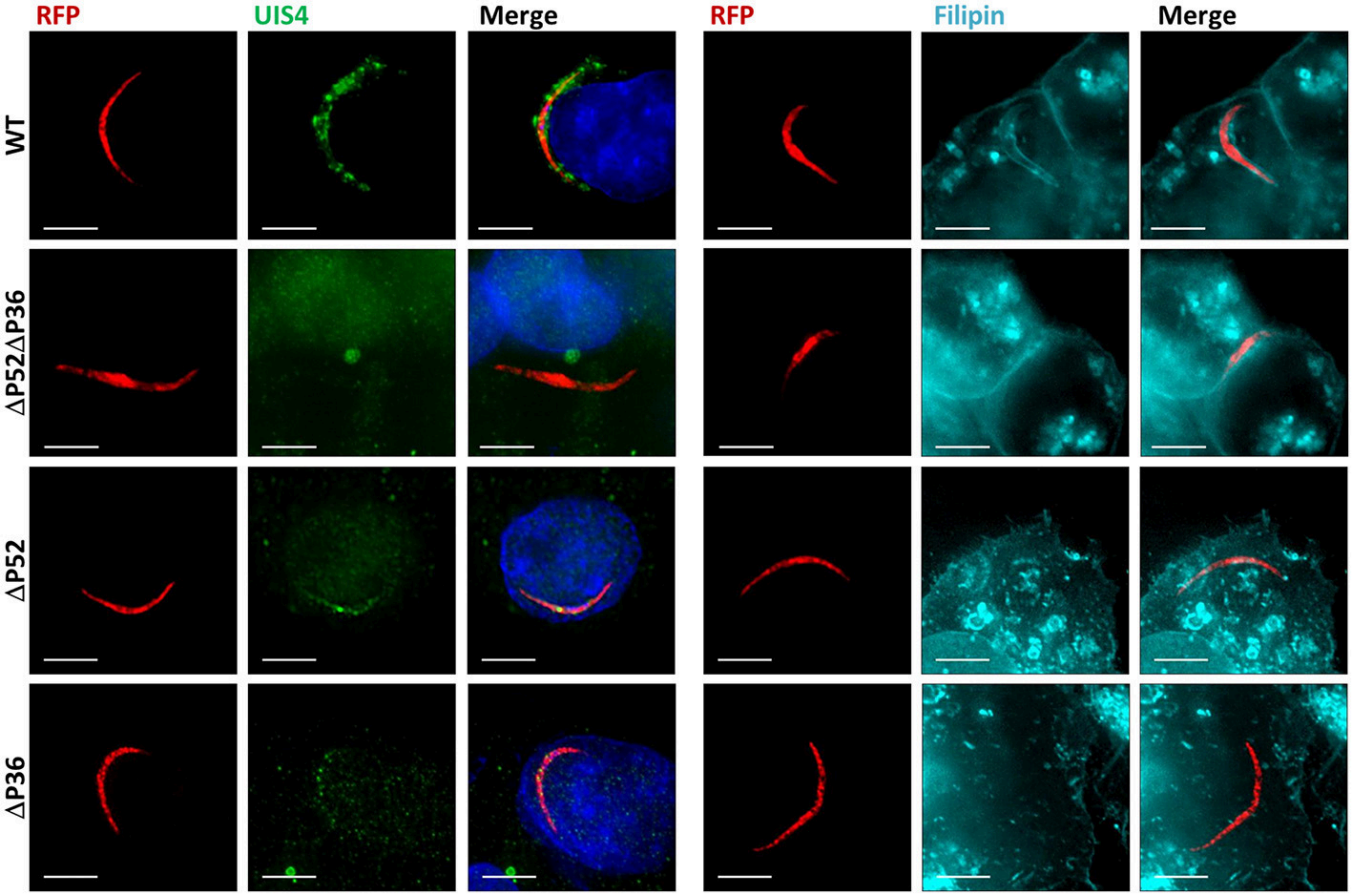
Both P36 and P52 are Required for the Formation of the Parasitophorous Vacuole. Representative fluorescence microscopy images of intracellular RFP-tagged salivary gland sporozoites in Hepa 1–6 cells (2 hpi) from two different experiments. **Left**: Labeling fixed samples with α-UIS4 antibodies shows the presence of a PVM in wild-type parasites but not in single or dual deletion *Py* Δ*P36*, Δ*P52*, and Δ*P52*Δ*P36* mutants. **Right**: Live staining with the sterol dye filipin confirms a PVM surrounding the wild-type parasites and the absence of a membrane for the mutant lines (Figure S2) (Scale bar: 5 μm).

It remains unknown whether the deletion of *P52* or *P36* alone results in the defective PV phenotype previously observed for Δ*P52*Δ*P36* sporozoites (Labaied et al., 2007; Ploemen et al., 2012). To address this question, RFP-tagged Δ*P36* and Δ*P52* single mutant parasites were allowed to infect Hepa 1–6 hepatoma cells for 2 h. Analysis of infected hepatocytes by IFA (Figure 1) using α-UIS4 antibodies as PVM markers showed the presence of an apparently normal PVM for wild-type parasites as indicated by circumferential UIS4 staining around intracellular sporozoites. However, no PVM was observed for the Δ*P52*Δ*P36* parasites as expected. Importantly, neither the Δ*P52* nor the Δ*P36* single knockout intracellular parasites showed a PVM; only residual signal within sporozoites was observed for UIS4, possibly indicating UIS4 that was not secreted from the parasite. Furthermore, staining with filipin, which fluorescently labels sterols (Figure 1, Figure S2), showed a hepatocyte-plasma-membrane-derived membrane surrounding wild-type intracellular parasites, but no membrane was observed around the Δ*P36* and Δ*P52* single knockouts or Δ*P52*Δ*P36* double knockout parasites, confirming the absence of a host-derived PVM.

### P36 and P52 localize to the sporozoite micronemes

*Plasmodium yoelii* parasites were genetically modified by double crossover recombination to substitute the expression of P36 with a version of P36 fused to an mCherry tag at the C-terminus (Figure S1B). P36^mCherry^ parasites successfully progressed through development within the mosquito vector, and an *in vivo* pre-patent period comparable to wild-type parasites was observed; *in vitro*, sporozoites displayed normal gliding motility (Figure S3) and were capable of invading hepatoma cells (Figure S4), indicating no significant negative impact on protein function derived from the fusion with the mCherry tag.

**Figure 2:**
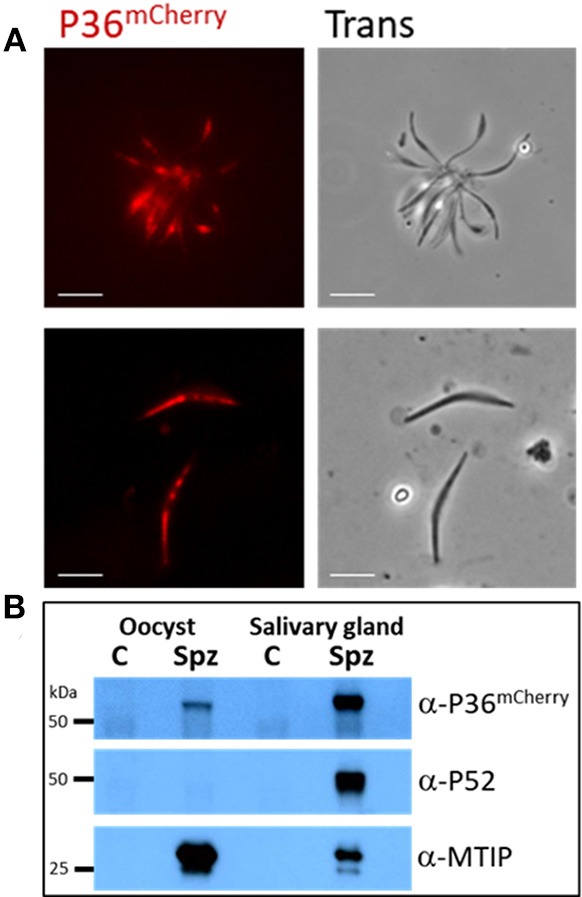
P36^mCherry^ Expression in *P. yoelii* Sporozoites. **(A)** Live fluorescence microscopy of freshly isolated oocyst (top) or salivary gland (bottom) sporozoites show expression of P36^mCherry^; transmitted light images are also shown. **(B)** Relative expression of P36^mCherry^ and P52 as compared to MTIP in oocyst and salivary gland sporozoites analyzed by western blot (1 M spz per lane). Membrane was incubated with α-mCherry (16D7) and donkey α-rat-HRP; stripped and incubated with α-PyP52 (13G10) and goat α-mouse-HRP; stripped and incubated with α-MTIP and goat α-rabbit-HRP as a loading control. Spz, P36^mCherry^ sporozoite lysate; C, uninfected midgut or salivary gland extracts used as control for nonspecific antibody binding; Oocyst spz from day 10 (Scale bar: 5 μm).

P36^mCherry^ was found to be expressed in both oocyst (day 10) and salivary gland-derived sporozoites by live fluorescence microscopy (Figure 2A) and confirmed by western blot analysis using α-mCherry (α-P36 antibodies (Supplementary Methods) were not functional in western blots) where single bands were observed for P36^mCherry^ and P52 (using α-P52 antibodies) approximately at the predicted molecular weights (MW) of 66 and 53 kDa, respectively (Figure 2B). The semi-quantitative densitometry analysis of the protein signals normalized to the signal of the inner membrane complex protein MTIP (myosin A tail domain interacting protein, 24 kDa), which is expressed in both sporozoite forms, indicated that P36^mCherry^ is expressed at significantly higher levels in salivary gland sporozoites as compared to oocyst sporozoites. P52, however, was only detected in salivary gland sporozoites (Figure 2B).

**Figure 3:**
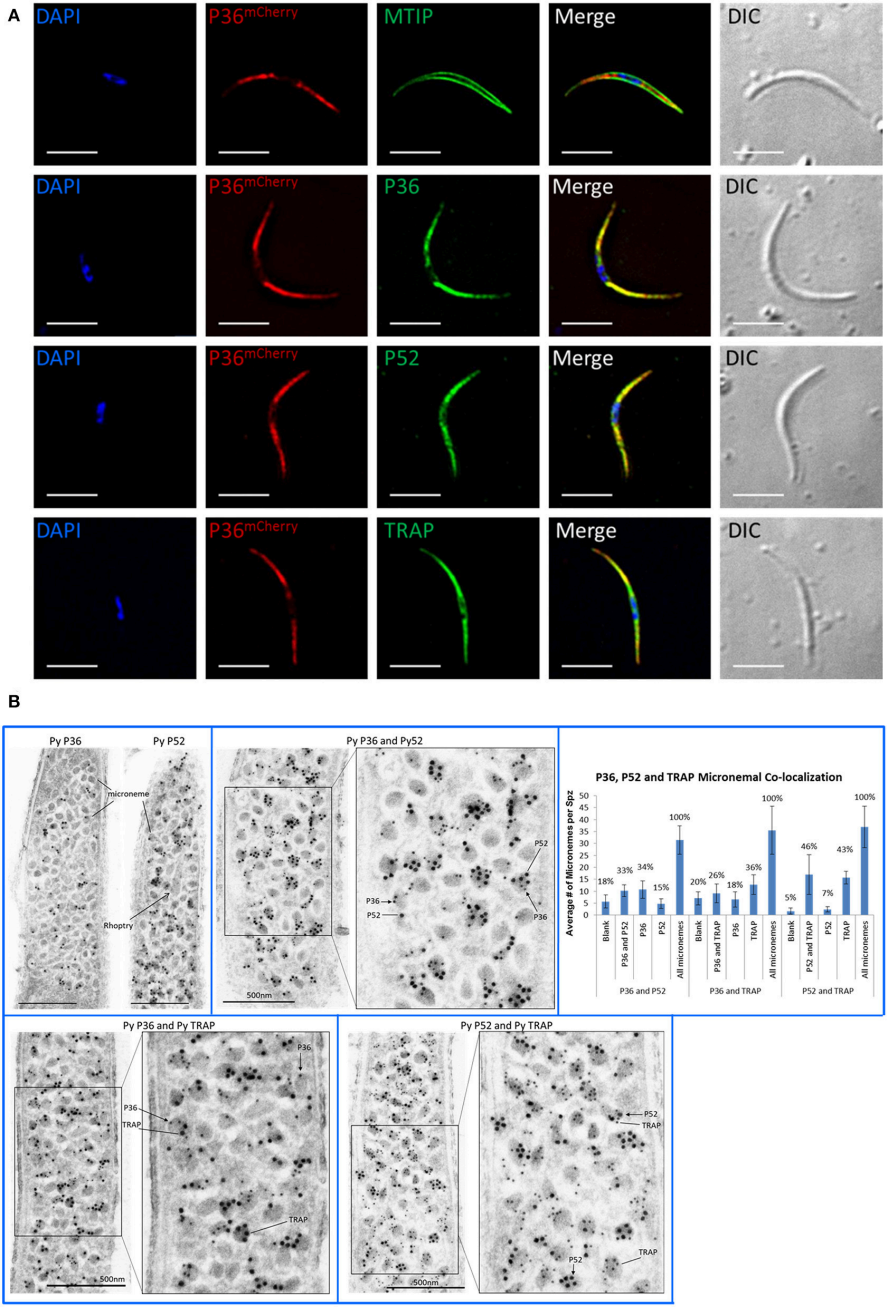
P36 and P52 are Localized in the Sporozoite Micronemes. **(A)** Representative immunofluorescence microscopy images of P36^mCherry^ salivary gland sporozoites showing the internal localization of P36^mCherry^ compared to the micronemal proteins P52 and TRAP and in contrast to the inner membrane complex protein MTIP. Second row shows signal co-localization with α-mCherry and α-PyP36 respectively. Nuclei were stained with DAPI. Primary antibodies used: rat α-mCherry, rabbit α-MTIP, mouse α-PyP36.1, mouse α-PyP52 (13G10), mouse α-PyTRAP.2. Secondary fluorescently-labeled antibodies: α-rat-AF^594^, α-rabbit-AF^488^, and α-mouse-AF^488^ (Scale bar: 5 um). **(B)** Representative electron microscopy images of P36^mCherry^ sporozoites show the co-localization of P36, P52 and TRAP in the micronemal organelles. Top left: P36^mCherry^ (α-PyP36.1) and P52 (α-P52-2D5) are shown in the micronemes of individual sporozoites (15 nm gold). Top right: dual labeling with rabbit α-PyP36 (S) and mouse α-PyP52 (2D5) (L). Bottom left: dual labeling with rabbit α-PyP36 (S) and mouse α-PyTRAP.2 (L). Bottom right: dual labeling with rabbit α-TRAP (S) and mouse α-PyP52 (2D5) (L). The chart shows the percentage of micronemes with single and dual labeling in the analyzed sporozoites (Figure S6). (S) = 10-nm gold; (L) = 15 nm gold (Scale bar: 500 nm).

Immunofluorescence microscopy of permeabilized P36^mCherry^ salivary gland sporozoites (Figure 3A) showed P36^mCherry^ labeling distributed in a cell-internal pattern typically observed for micronemal localization and different from the inner membrane complex pattern observed with MTIP. Sporozoites dually labeled with α-mCherry and α-P36 antibodies displayed signal overlap confirming the P36^mCherry^ pattern corresponds to P36. Similar signal overlap was observed for sporozoites dually labeled with α-mCherry and α-P52 or α-TRAP (thrombospondin-related anonymous protein), both proteins previously reported to be localized in the micronemes (Rogers et al., 1992; Gantt et al., 2000; Ishino et al., 2005; VanBuskirk et al., 2009), suggesting the co-localization of P36 with P52 and/or TRAP in the micronemal organelles. Equivalent staining patterns were found for P36, P52, and TRAP in wild-type sporozoites (Figure S5).

To determine the precise subcellular localization of P36, and to confirm the localization of P52 with higher resolution than previously reported (Ishino et al., 2005), immunolabeled P36^mCherry^ salivary gland sporozoite ultrathin sections were analyzed by electron microscopy (Figure 3B, Figure S6). P36 and P52 were clearly observed predominantly contained within the micronemal organelles in single-labeled sporozoites (Figure 3B). In addition, dual labeling experiments using 10-nm gold and 15-nm gold particles allowed, for the first time, the observation of the co-localization of P36 and P52, P36 and TRAP, and P52 and TRAP within the same micronemes. Interestingly, not all micronemes within a sporozoite were uniformly labeled; there were populations of unlabeled (blank), single labeled, and double labeled micronemes within each sporozoite for every label combination as shown in Figure 3B. For P36 and P52 labeling, about a third (33%) of the counted micronemes were dually labeled, and a similar number were labeled only with P36 (34%); however, only 15% of the total number of micronemes was singly labeled with P52 (Figure 3B, Figure S6). In other words, approximately half of the micronemes (*F* = 0.49) labeled with P36 were also labeled with P52, while more than two thirds (*F* = 0.68) of all P52-labeled micronemes were simultaneously labeled with P36. For P36 and TRAP, out of all micronemes labeled with TRAP, 42% also contained P36; and about 58% of all P36-labeled micronemes were also labeled with TRAP. For P52 and TRAP, there was a similar percentage of single and double labeled micronemes containing TRAP; however, the majority of all P52-labeled micronemes (nearly 88%) were also labeled with TRAP. Rather than reflecting a random distribution, these findings are indicative of an intrinsic higher probability for P52 to be localized in micronemes containing either TRAP or P36, instead of being found individually. Therefore, a mathematical correlation should exist between the probabilities of finding P52 co-localized [(P52 + P36) and (P52 + TRAP)] and the probability of finding TRAP and P36 in a given microneme. Accordingly, and taking the observed frequencies (F) as probabilities (p), p[P52 + P36]^*^ p[P52 + TRAP] = p[P36 + TRAP] resulting in (0.68^*^0.875) = 0.595, which is remarkably close to the experimentally observed frequency (*F* = 0.58) for finding P36 and TRAP together, suggesting a real tendency for P52 to be found co-localized. Triple labeling experiments were attempted, however none of the rat α-mCherry antibodies tested worked well for this purpose.

### P36 interacts with P52 but not with TRAP

**Figure 4:**
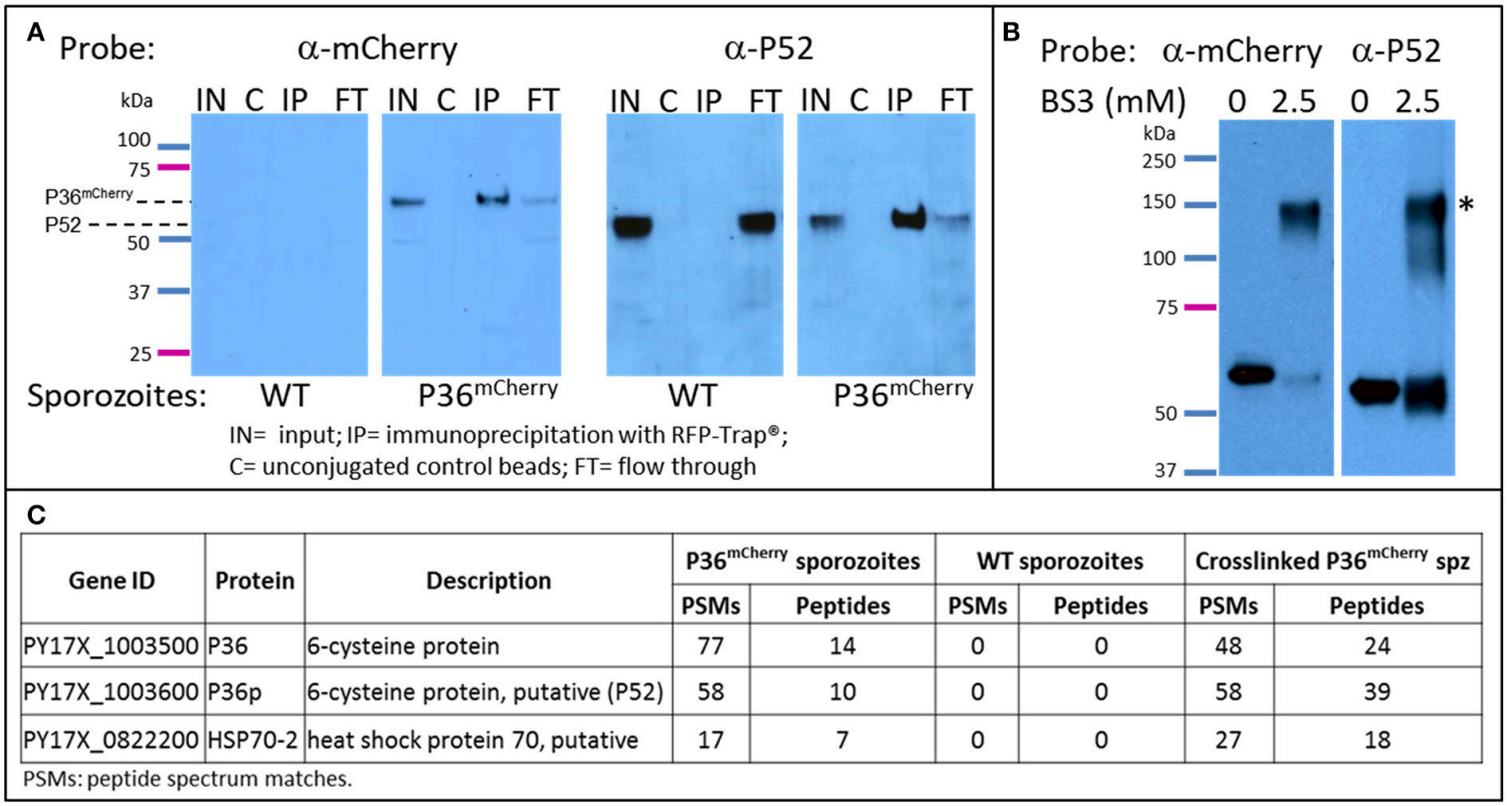
P36 and P52 are Interacting Partners. Western blot analysis of: **(A)** The immunoprecipitation of P36^mCherry^ (IP) from sporozoite lysates (labeled as input = IN) showing co-precipitation of P52. Wild-type sporozoite lysates were used as a control for nonspecific binding (left panels). **(B)** Chemically crosslinked (2.5 mM BS3) P36^mCherry^ sporozoite lysate showing a higher MW band (*) recognized by both α-mCherry and α-P52 antibodies in comparison with non-crosslinked lysate (0.5 M spz per lane). Blots for both **(A,B)** were first incubated with rabbit α-mCherry primary antibody and α-rabbit-HRP, stripped, and re-incubated with α-P52 (13G10) and α-mouse-HRP. **(C)** Liquid chromatography-mass spectrometry analysis results showing P36 and P52 were the most abundant sporozoite proteins recovered from the IP of P36^mCherry^ from non-crosslinked and crosslinked lysates and were not detected in the wild-type samples. The total number of peptide spectrum matches (PSMs) and unique peptides identifying the proteins in each experiment is given. PSMs and peptides were only counted for peptides that were not shared with non-*Plasmodium* proteins, e.g., mosquito.

To explore the possible interacting partners of P36 within the sporozoite, P36^mCherry^ was immunoprecipitated (IP) from salivary gland sporozoite lysates using RFP-Trap®, technology based on alpaca nanobodies with remarkable affinity and specificity to the mCherry tag (Kd = 5 nM, Chromotek). Wild-type sporozoite lysates were used in parallel as a control for non-specific binding. Analysis of the immunoprecipitated samples by western blot (Figure 4A) probed with an α-mCherry antibody showed no non-specific interaction when using wild-type sporozoite lysates. However, one prominent protein band was observed near the expected MW (66 kDa) for P36^mCherry^ in the input (IN) and in the RFP-Trap® precipitated sample (IP). The unconjugated bead control (C) did not show a signal, as expected, and only a faint band was seen in the flow through (FT), indicating most of P36^mCherry^ was successfully immunoprecipitated. Probing with α-P52 antibody after stripping revealed the presence of P52 in the input (IN) of both wild-type and P36^mCherry^ sporozoites near the expected MW (53 kDa), and an equal intensity band for the wild-type flow through was observed as there was no depletion from incubating with RFP-Trap®. In the P36^mCherry^ sporozoite sample, P52 was observed with the same pattern as P36^mCherry^ with a strong band in the immunoprecipitated sample (IP), only a faint band left in the flow through (FT) sample, and no signal for the unconjugated bead control (C), indicating a specific affinity of P52 for P36^mCherry^. Probing with α-TRAP antibodies after stripping, as an additional control, did not show a signal for any of the immunoprecipitated (IP) samples (data not shown).

To further investigate the P52-P36 interaction, P36^mCherry^ sporozoite lysates were chemically crosslinked using 2.5mM bis[sulfosuccinimidyl] suberate (BS3) (Figure 4B) and analyzed by western blot, including non-crosslinked lysate as a comparison. A higher MW signal (denoted with^*^) was observed for the crosslinked lysates when probing with α-mCherry indicating that P36^mCherry^ is part of a protein complex with an apparent MW of ~120–150 kDa when crosslinked. The detected signal was within range of the expected MW of the P36^mCherry^-P52 complex (119 kDa) considering that a more native-like conformation should be preserved by the crosslinker. Furthermore, stripping and re-incubating the blot with α-P52 confirmed the presence of P52 within the same crosslinked complex. An equivalent shift in MW was also observed for P52 using crosslinked lysates from wild-type sporozoites (data not shown).

Immunoprecipitated P36^mCherry^ and co-precipitated protein partners were analyzed in more detail by mass spectrometry comparing against the IP of the wild-type lysate made with the same number of sporozoites; both lysates were previously incubated with unconjugated beads to assess non-specific binding. The relative abundance of proteins detected from test and control samples was assessed by spectral counting. The *Plasmodium* proteins exhibiting the highest enrichment relative to control were P36^mCherry^, P52, and HSP70-2/BiP (Figure 4C and Table S3). None of these were detected in the wild-type IP or the IP of P36^mCherry^ sporozoites with unconjugated beads. No other *Plasmodium* proteins were appreciably enriched relative to controls (Table S3). In the BS3-crosslinked sample, P36 and P52 were the highest abundance *Plasmodium* proteins. When intra-sample protein abundances were compared by normalizing the number of identifying PSMs to protein length (i.e. spectral abundance factor, Zybailov et al., 2006), P36 and P52 were present at approximately 1:1 abundance ratio. Several other *Plasmodium* proteins were identified at abundances above background, including HSP70-2/Bip, but at more than 2-fold lower abundance than P36 and P52.

To exclude the remote possibility of P52 binding to the mCherry tag instead of P36, an inverse pulldown was performed starting with P52^myc^ sporozoites, genetically modified to express a second copy of P52 fused to a c-terminal 4xMyc tag (Supplementary Methods and Figure S1C), and immunoprecipitated with a Myc-Trap®. Western blot analysis probing with α-Myc showed the successful IP of P52^myc^ with no non-specific binding for *Py* GFP-Luc sporozoite lysates used as wild-type controls (Figure S7). The P52^Myc^ and wild-type IPs were subsequently analyzed by mass spectrometry. The *Plasmodium* proteins exhibiting the highest enrichment relative to control were P52, HSP70-2/BiP, and P36. P52 and P36 were absent in the control IP. HSP70-2/BiP was identified at low abundance in the control (three total PSMs from three unique peptides) but was high-abundance in the test IP (78 PSMs from 25 unique peptides). Other *Plasmodium* proteins in the test IP were less abundant relative to the control (Figure S7 and Table S3). These results confirm the specific binding of P36 to P52 and a possible interaction with HSP70-2/BiP. Although TRAP was also detected in these experiments, its abundance was similar between test samples and controls indicating non-specific interactions (Table S3).

### P36 is secreted by activated sporozoites

**Figure 5:**
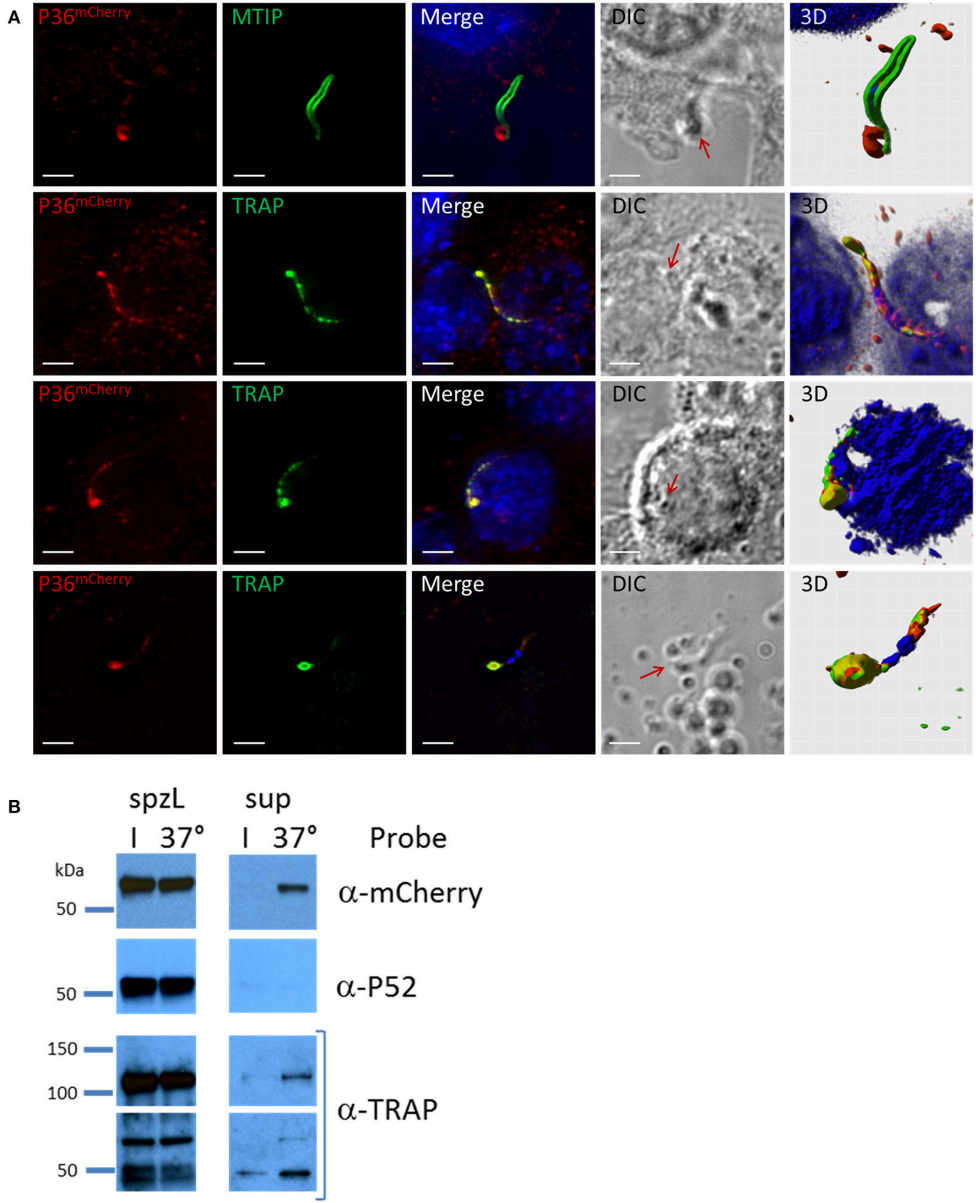
P36 is Secreted Upon Sporozoite Activation. **(A)** Representative immunofluorescence microscopy images of activated sporozoites within the context of a Hepa1-6 cell invasion assay (90 min) showing the accumulation of P36^mCherry^ at the apical end of the sporozoite similar to the secreted protein TRAP, and in contrast to MTIP. The last row shows an isolated sporozoite that appears attached to the glass with an accumulation of P36^mCherry^ at the apical end surrounded by a TRAP rim, suggesting of the movement of TRAP toward the rear end. All other rows show sporozoites in contact with or within a cell. Nuclei were stained with DAPI. Primary antibodies used: rat α-mCherry, rabbit α-MTIP and mouse α-PyTRAP.2. Secondary fluorescently-labeled antibodies: α-rat-AF^594^, α-rabbit-AF^488^ and α-mouse-AF^488^ (Scale bar: 5 um). Red arrows point toward protein concentration on DIC images. 3D volume visualization was done with Imaris. **(B)** Western blot analysis shows the secretion of P36^mCherry^ into the supernatant following sporozoite activation at 37°C similarly to the previously reported secretion of TRAP. Sporozoites were incubated in PBS either on ice (I) or at 37°C for 40 min. Sporozoites were separated from the total supernatant (sup), pelleted, and boiled in SDS buffer to create whole sporozoite lysate (spzL) (0.8 M spz per spzL lane). Blots were first incubated with rabbit α-mCherry primary antibody and α-rabbit-HRP, stripped and re-incubated with α-P52 (13G10) and α-mouse-HRP, stripped again and re-incubated with rabbit α-TRAP and α-rabbit-HRP.

Analysis of P36^mCherry^ salivary gland sporozoites by IFA in the context of Hepa 1–6 cell invasion (Figure 5A) revealed the presence of an accumulation of P36^mCherry^ protein toward one end of the sporozoite, reminiscent of the previously reported secretion of TRAP (Rogers et al., 1992; Gantt et al., 2000). This concentrated protein appeared to be released from the sporozoite (Figure 5A, top row) when contrasted with the MTIP-labeled inner membrane complex, and it consistently co-localized with unprocessed, full-length TRAP (lower rows). The staining pattern for both P36^mCherry^ and TRAP regularly overlapped at what appeared to be progressive stages of secretion where the concentration of the proteins at the apical end became more prominent as the internal micronemal signal diminished. A single exception to this pattern is shown in the last row, where an isolated sporozoite that was seemingly attached to the glass slide (no cell was observed nearby) displayed a bulk center-concentrated signal for P36^mCherry^ and an outer rim-like signal for TRAP (Figure S8A), possibly capturing the moment at which membrane-anchored TRAP begins translocation toward the posterior end of the sporozoite while P36^mCherry^ is secreted/released. Although the α-P36 and α-P52 antibodies showed a very high background staining for Hepa 1–6 cells, signal overlap for P36 and P36^mCherry^, and P52 and P36^mCherry^, was detected (Figure S8B), displaying the same apical end concentration pattern.

To explore secretion of P36 and P52 in greater detail, the same number of P36^mCherry^ sporozoites were incubated on ice or at 37°C and pelleted by centrifugation. Analysis of whole sporozoite pellets compared to total supernatants by western blot (Figure 5B) revealed the presence of TRAP in full-length and processed form as a positive control, and also the secretion of P36^mCherry^ at 37°C. Interestingly, P52 was not found in the supernatant under these conditions.

## Discussion

*Plasmodium* sporozoite infection of the liver is necessary for the malaria parasite life cycle to initiate in the mammalian host. Deletion of *P36* and *P52* from parasite genomes has proven to drastically impact sporozoite infectivity and this is linked to the inability of knockout sporozoites to productively invade hepatocytes and initiate exoerythrocytic schizogony. Our *in vivo* infection results measuring the onset of blood stage patency after sporozoite inoculation showed severe defects of both the *Py* Δ*P52* and Δ*P36* individual gene deletion strains as well as the Δ*P52*Δ*P36* dual gene deletion strain, although the latter showed a slightly more severe phenotype as reported previously (Labaied et al., 2007). The loss of *in vivo* infectivity has been attributed to the absence of a PVM, demonstrated by the electron microscopic analysis of *Py* and *Pb* dual deletion Δ*P52*Δ*P36* mutants found free within the host hepatocyte cytoplasm or in the nucleus, without a protective PVM, in *in vitro* hepatocyte infections (Labaied et al., 2007; Ploemen et al., 2012). However, in these experiments, the simultaneous deletion of *P36* and *P52* did not allow the assessment of the individual contributions of the two proteins in the establishment of a PVM. Our results demonstrate that both proteins are necessary early in the process of formation of a functional PVM as neither the *Py* Δ*P52* nor the Δ*P36* single knockout intracellular parasites showed circumferential labeling for the PVM protein UIS4, pheno-copying the Δ*P52*Δ*P36* parasites. In some cases, only a residual signal was observed contained within the sporozoite. This likely corresponds to UIS4 protein within secretory organelles of the intracellular sporozoite that has not been secreted or remnants thereof. The PVM is enriched with cholesterol originating from the host cell which can be readily labeled with the sterol-binding dye filipin (Bano et al., 2007). Here we showed that, while a PVM is clearly observed in intracellular *Py* wild-type sporozoites, intracellular *Py* Δ*P52* and Δ*P36* individual gene deletion parasites (as well as the Δ*P52*Δ*P36* parasites) lacked circumferential filipin staining, further showing the inability of single gene knockouts to form a PVM. Sporozoites also traverse host cells but in transient vacuoles (TV) formed without rhoptry secretion or membrane remodeling before committing to productive invasion, which could confound the detection of true PVMs in which the parasites are ensconced. However, our analysis of UIS4, which is only inserted into the PVM but not the TV, as well the time of analysis of 2 h post sporozoite infection, when cell traversal has mostly ceased, make this unlikely (Risco-Castillo et al., 2015). Together our results indicate that P36 and P52 individually have an important role within the same pathway leading to the induction of PVM formation during productive invasion.

Earlier transcriptional analyses showed the presence of P36 and P52 transcript already in oocyst sporozoites (Kappe et al., 2001; Lakshmanan et al., 2015), however, both proteins have been shown to be expressed at levels detectable by western blot only in *P. berghei* salivary gland sporozoites (Ishino et al., 2005). Our results showed a fluorescent signal for *Py* P36^mCherry^ already in day-10 oocyst sporozoites, and expression of P36^mCherry^ was confirmed by western blot, albeit at a lower signal intensity than in salivary gland sporozoites. The use of a mCherry tag together with more potent new reagents possibly magnifies the signal, expanding the limit of detection compared to previous reports. In our study, *Py* P52 expression was only observed in salivary gland sporozoites.

Host-cell invasion studies in *Toxoplasma* and *Plasmodium* merozoites indicate there are three sets of apical organelles in invasive stages: micronemes, rhoptries, and dense granules, which are progressively released early during and after successful host cell invasion. Micronemes, the first set of organelles to be released, store proteins essential for initiation and progression of invasion, and in *Plasmodium* merozoites and *Toxoplasma* have been shown to secrete proteins critical for the formation of the moving junction (Dubremetz et al., 1998; Carruthers et al., 1999; Blackman and Bannister, 2001; Soldati et al., 2001; Sharma and Chitnis, 2013). The thrombospondin-related anonymous protein, TRAP, is one of the best-studied sporozoite proteins because of its multiple and essential roles in motility and cell invasion, being the model protein for micronemal localization and secretion in *Plasmodium* (Rogers et al., 1992; Sultan et al., 1997; Menard, 2000, 2001). The creation of the *Py* P36^mCherry^ transgenic parasites allowed us to unequivocally localize P36, and partially co-localize it with P52 within the micronemal organelles with a pattern reminiscent of the micronemal protein TRAP. The validation of the observed localization patterns was also possible in wild-type sporozoites as α-P36 antibodies, capable of recognizing native P36 in IFA, became available later in our studies. Little is known about the cargo identity of sporozoite micronemes; whether all micronemes are uniformly loaded with the same protein contents, or if different populations of micronemes with distinct cargo do exist is unknown as no isolation and direct analysis of *Plasmodium* sporozoite micronemes has been reported (Blackman and Bannister, 2001). However, the presence of different microneme subsets or subcompartments has been shown for *Toxoplasma* tachyzoites (Kremer et al., 2013). Using TRAP as a reference protein, we investigated if P36 and P52 were found within the same micronemes; therefore, we analyzed the P36^mCherry^ sporozoites by immune electron microscopy, resulting in the unambiguous localization of P36 and P52 in the micronemes. Furthermore, analysis of dually-labeled sporozoites showed micronemes containing P36 and P52, P36 and TRAP, and P52 and TRAP alongside micronemes labeled only for one or the other protein, confirming that these proteins can indeed be found frequently co-localized within the same physical compartment. The mixed single/double label pattern seen in any given section could possibly be explained by the limitations of observing a three-dimensional microneme in a 2-dimensional section, appearing to be only singly labeled in the XY planes while the second protein might be concealed in the unseen Z plane. Intriguingly, systematic counting of multiple dually-labeled sporozoite sections indicates that while P36 or TRAP labeling seems to be relatively equally distributed into single and double stained micronemes, P52 appears to have a higher tendency to be found in micronemes containing TRAP or P36, rather than individually. Yet, we found strong evidence that P36 and P52 directly interact, but we did not find a specific interaction of either protein with TRAP as discussed below. In general, these findings suggest the presence of different populations of micronemes within sporozoites, a discovery that if confirmed, would bring us closer to a deeper understanding of the process of regulation of different steps in hepatocyte invasion and this deserves further exploration.

P36 and P52 belong to the *Plasmodium* s48/45 protein family which comprises 14 members. Four of these proteins, P48/45-P230 and P12-P41, have already been reported to work in pairs, where the first protein of each pair is predicted or known to be GPI-anchored (Kumar, 1987; Taechalertpaisarn et al., 2012; Parker et al., 2015; Arredondo and Kappe, 2016). Given the propensity of s48/45 proteins to function in pairs, and the reported phenotypes for P36 and P52, apparently within the same functional pathway, we hypothesized that these proteins also work as a pair. Our analysis of P36^mCherry^ immunoprecipitations consistently showed its association with P52. Furthermore, a protein complex with a MW approximately the size of P36^mCherry^ and P52 combined was clearly detected containing both proteins when the lysate was chemically crosslinked. An inverse pulldown using *Py* P52^Myc^ sporozoites confirmed the specificity of the P36-P52 interaction. Our results demonstrate for the first time that P36 and P52 form a complex, which is directly in line with our findings that both proteins work within the same functional pathway. Interestingly, while P52 was the most abundant protein partner in the immunoprecipitations, HSP70-2 was also found in high proportion in the complex by mass spectrometry. HSP70-2, also known as BiP, is a conserved member of the heat-shock chaperone family, expressed in all stages of the *Plasmodium* life cycle, and reportedly localized in the endoplasmic reticulum (ER) and Maurer's clefts in blood stages (Kumar et al., 1991; Vincensini et al., 2005; Shonhai et al., 2007; Aurrecoechea et al., 2009). The function of HSP70-2/BiP is unknown; however, potential associations with an array of proteins in different pathways, including a protein exported to the erythrocyte has been reported (LaCount et al., 2005), suggesting possible chaperone capabilities. A potential role as a chaperone for P36-P52 or other secreted proteins is supported by the observation that HSP70-2/BiP has been identified as putatively surface-exposed by proteomic analyses of surface-biotinylated sporozoites (Swearingen et al., 2016, 2017). Additional studies will be necessary to elucidate the role of HSP70-2/BiP in the P36-P52 complex. The P36-P52 interaction appears to be robust as it persists through extensive washing; however, other more transient or weaker interactions, possibly lost with our standard procedures, may have been preserved by chemical crosslinking. Analysis of the crosslinked samples by western blot showed only one major higher-MW signal corresponding to the P36-P52 complex; however, the α-P52 antibody also showed weaker signals below the main P36-P52 band, which might be indicative of transient interactions with other proteins. Mass spectrometry analysis of the crosslinked samples showed other lower-abundance potential interactions such as SIAP-1, a protein reported to function in oocyst sporozoite egress and salivary gland invasion (Engelmann et al., 2009), however, further investigation would be necessary to determine the biological significance of these low abundance associations.

At the time of invasion, proteins stored in the micronemes are secreted to establish contact with the corresponding receptors. Microneme secretion is a temperature dependent process and it is regulated by the mobilization of parasite intracellular Ca^2+^ (Carruthers and Sibley, 1999; Carruthers et al., 1999). Following activation of the sporozoite, micronemes polarize to the apical end and exocytose by fusing with the plasma membrane, thereby discharging their protein contents. In the case of TRAP, the protein remains associated with the membrane upon secretion via its transmembrane domain and is subsequently translocated toward the rear end of the sporozoite, eventually being cleaved and shed (Menard, 2001; Soldati et al., 2001). Activated sporozoites analyzed by IFA exhibited a cap-like accumulation of TRAP at the apical end (Rogers et al., 1992; Gantt et al., 2000) that progressed into surface rings during gliding motility (Kappe et al., 1999; Menard, 2000). Our IFA analysis of P36^mCherry^ sporozoites that were seemingly in contact with cells also showed an accumulation of P36^mCherry^ toward one end and its co-localization with the TRAP cap. For both proteins, strong accumulation of protein at the apical end seemed to be more often linked with less protein observed within the sporozoite, suggesting an active and progressive movement and release of micronemal proteins toward the apical end. Analysis of the apical localization of the P36^mCherry^ in contrast with MTIP labeling at the inner membrane complex suggests P36^mCherry^ is being secreted from the sporozoite. In addition, we were able to observe a clear difference in localization once TRAP appeared to initiate rear movement forming a ring structure at the apical tip of the sporozoite, while the P36^mCherry^ signal remains in bulk; this finding is in agreement with P36 not having a transmembrane domain for insertion into the membrane. Despite the limitations imposed by the high signal background detected on cells with our α-P36 and α-P52 antibodies, we did observe apical signal overlap of α-mCherry with α-P36 and also co-localization with P52, verifying the cap pattern corresponds to P36 and suggesting the possible secretion of P52. Prompted by these observations we proceeded to directly analyze sporozoite secretion of P36 and P52. Induction of TRAP secretion from sporozoites has been previously shown following incubation at 37°C (Bhanot et al., 2003). Likewise, incubation of P36^mCherry^ sporozoites at 37°C was sufficient to induce the secretion of P36^mCherry^ into the supernatant. Interestingly, P52 was not found in the secretion supernatant of sporozoites activated under any method tested, including temperature change or incubation with FBS or Ca^2+^ ionophore (not shown) (Carruthers and Sibley, 1999; Carruthers et al., 1999; Brown et al., 2016). P52 is predicted to be GPI-anchored; therefore, it would be expected to remain associated with a membrane. *Pb* P52 was originally described as being secreted and retained onto the sporozoite surface upon gliding (Ishino et al., 2005); however, we were unable to reproduce these findings using available reagents. It is possible that P52 is processed during translocation, losing the epitope recognized by our P52 mAb and preventing the detection of P52 on the surface of the parasite. Determining the fate of P52 will require further exploration.

**Figure 6:**
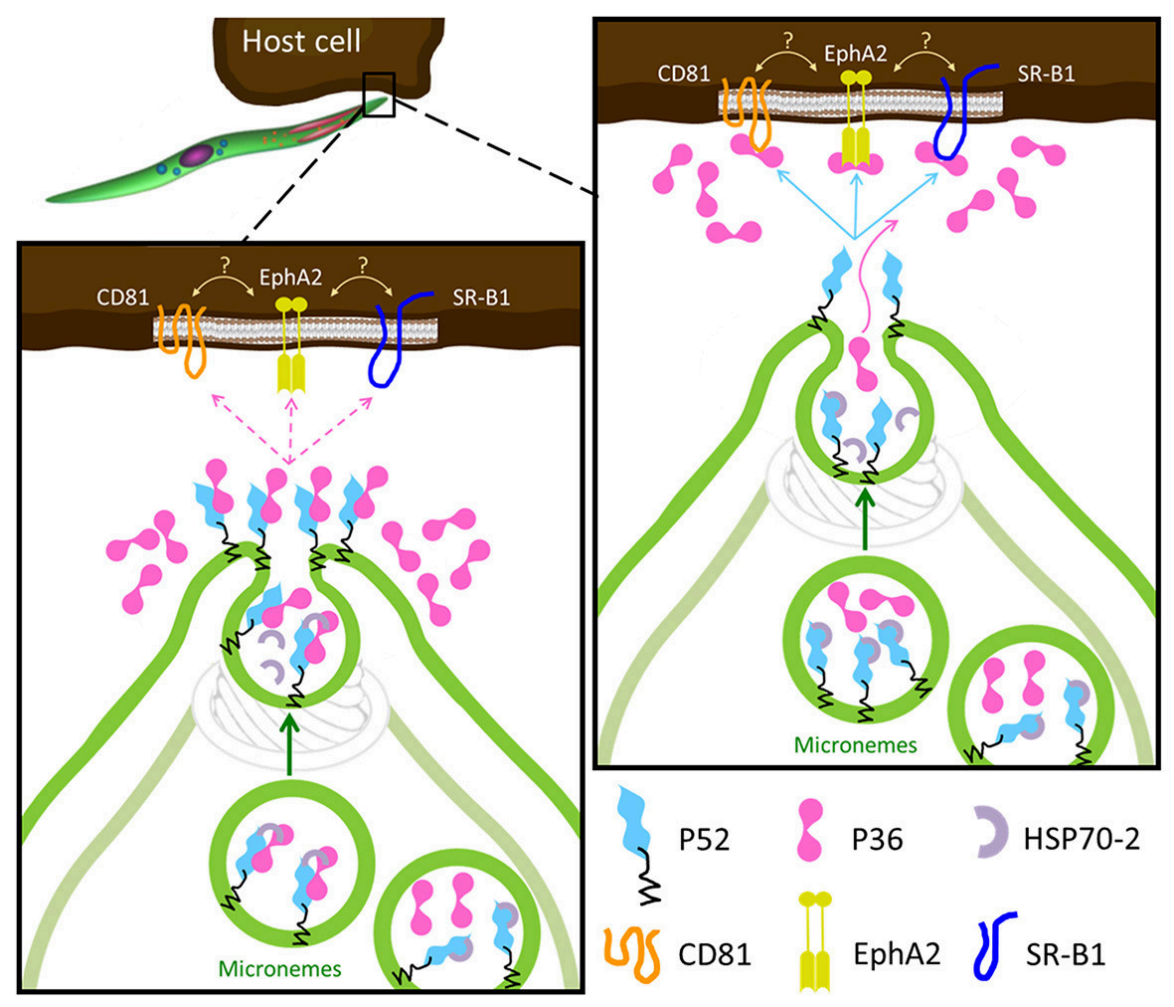
Proposed Models for the Role of P36 and P52 Leading to Host Receptor Engagement. In both models the GPI-anchored P52 serves as a scaffold for the interaction of P36 with the proposed host receptors. **Left**: upon activation, P36 and P52 assemble into a likely chaperoned complex within the micronemes. As micronemal secretion takes place, the complex is brought to the apical end, the chaperone is released, and P52 displays P36 aiming to engage the host receptor; if a successful interaction takes place then the formation of the PVM is induced and invasion proceeds, if not, P36 is released into the surroundings. **Right**: the second model is more consistent with the robust interaction between P36 and P52 and with the soluble secretion of P36. In this model, P36 and P52 would be kept separated by the chaperone within the microneme, P36 is then released alone during microneme secretion to establish an interaction with the corresponding host receptor; then, P52 at the apical end (or even translocated to the surface) engages host-bound P36 inducing PVM formation and proceeding with productive invasion.

Despite both P36 and P52 being essential for productive hepatocyte invasion, recent studies suggest that only P36 might interact with the host cell. Previous indirect evidence was reported for the interaction of P36 with the host cell receptors EphA2, CD81 and SR-B1. All three host cell receptors are required for productive invasion to various degrees and, in the case of EphA2 and CD81, for PVM formation (Kaushansky et al., 2015; Manzoni et al., 2017). However, the contribution of EphA2 in these processes was brought into question in a recent study based mainly on the reduction of EphA2 expression levels using siRNAs (Langlois et al., 2018). The findings presented here show for the first time that both P36 and P52 are individually necessary for the formation of the replication-permissive PVM, that P36 is localized (and frequently co-localized with P52) in the sporozoite micronemes and subsequently secreted, and that both proteins form a complex in sporozoites. Combining the host-receptor studies and our new findings, we propose two possible models for the roles of P36 and P52 in invasion (Figure 6). In both models the GPI-anchored P52 would function as a scaffold and form a complex with P36 for their interaction with a hepatocyte receptor. A similar scaffolding role has been suggested for the GPI-anchored P12 and P48/45 proteins, also from the s48/45 family (Arredondo and Kappe, 2016). In a conservative first model, P36 and P52 would form a complex within the micronemes possibly assisted and/or protected by the HSP70-2/Bip chaperone, and as secretion takes place, the complex would be brought to the apical end, get de-protected and, upon microneme fusion with the sporozoite plasma membrane (PM), P52 will become PM-anchored and display P36 to establish a connection with the hepatocyte surface. If a successful interaction takes place, then productive invasion with PVM formation proceeds. Unbound P36 would be released. In the second model, P36 and P52 would be kept separated within the microneme by HSP70-2/Bip, P36 would be released alone during secretion to find and bind its corresponding host receptor; then, upon insertion into the PM, P52 would establish an interaction with host-bound P36 initiating productive invasion. A similar mechanism has already been reported for the AMA1-RON2 protein complex that is assembled during merozoite invasion of the erythrocyte for the formation of the moving junction (Besteiro et al., 2011). Determining whether the same strategy has been in fact exploited by the sporozoite during hepatocyte invasion and, providing a detailed description of the P36-P52 complex assembly along with the mechanisms of action of these two proteins will require further and meticulous investigation of the spatio-temporal dynamics of P36 and P52 in the context of host cell invasion.

## Author contributions

Conceptualization: SA, SM, AV, and SK; formal analysis: KS; funding acquisition: KS, NS, RM, and SK; investigation: SA, KS, TM, RS, DD, AH, SM, and MT; methodology: SA, KS, TM, RS, NC, SM, AV, and MT; resources: BO, VV, NK, TI, NS, and WB; supervision: NS, RM, and SK; visualization: SA, KS, and MT; writing-original draft: SA, KS, RS, VV, and AV; writing-review and editing: SK.

### Conflict of interest statement

The authors declare that the research was conducted in the absence of any commercial or financial relationships that could be construed as a potential conflict of interest.

## Abbreviations

ABC: ammonium bicarbonate
ACN: acetonitrile
BS3: bis[sulfosuccinimidyl] suberate
CD81: cluster of diferentiation 81
CID: collision-induced dissociation
DAPI, 4′: 6′-diamidino-2-phenylindole
DMEM: Dulbecco's modified Eagle media
EphA2: Ephrin type-A receptor 2
ER: endoplasmic reticulum
FA: formic acid
FDR: false discovery rate
GAP: genetically attenuated parasite
GPI: glycosylphosphatidylinositol
HCD: higher-energy collision-induced dissociation
HRP: horseradish peroxidase
I.D.: inner diameter
IFA: immunofluorescence assay
IP: immunoprecipitation
MTIP: myosin A tail domain interacting protein
MW: molecular weight
NSP: number of sibling peptides
O.D.: outer diameter
*P*.: *Plasmodium*
*Pb*: *Plasmodium berghei*
PFA: paraformaldehyde
PM: plasma membrane
PSM: peptide spectrum matches
PV: parasitophorous vacuole
PVDF: Polyvinylidene fluoride
PVM: parasitophorous vacuole membrane
*Py*: *Plasmodium yoelii*
RFP: red fluorescent protein
siRNAs: small interfering RNA
SR-B1: Scavenger receptor class B type 1
TBST, Tris buffered saline: 0.1% Tween-20
TFA: trifluoroacetic acid
TPP: Trans Proteomic Pipeline
TRAP: thrombospondin-related anonymous protein
TV: transient vacuole
UIS4: upregulated in infectious sporozoites gene 4.
