# Supplementary material for: The Micronemal Plasmodium Proteins P36 and P52 Act in Concert to Establish the Replication-Permissive Compartment Within Infected Hepatocytes
Source: Front Cell Infect Microbiol. 2018 Nov 27;8:413. doi: 10.3389/fcimb.2018.00413 (PMC6280682; doi:10.3389/fcimb.2018.00413)
Supplement: Supplementary file 1 [file Data_Sheet_1.pdf]

Supplementary Material

The micronemal *Plasmodium* proteins P36 and P52 act in concert to establish the replication-permissive compartment within infected hepatocytes

Silvia A. Arredondo[1], Kristian E. Swearingen[2], Thomas Martinson[1], Ryan Steel[1], Dorender A. Dankwa[1], Anke Harupa[1], Nelly Camargo[1], William Betz[1], Vladimir Vigdorovich[1], Brian G. Oliver[1], Niwat Kangwanrangsan[4], Tomoko Ishino[3], Noah Sather[1], Sebastian Mikolajczak[1], Ashley M. Vaughan[1], Motomi Torii[3], Robert L. Moritz[2], Stefan H. I. Kappe[1*]

[1]Center for Global Infectious Disease Research, Seattle Children's Research Institute, Seattle, Washington, United States of America, [2]Institute for Systems Biology, Seattle, Washington, United States of America, [3]Department of Molecular Parasitology, Proteo-Science Center, Ehime University, Shitsukawa, Toon, Ehime, Japan, [4]Department of Pathobiology, Faculty of Science, Mahidol University, Ratchathewi, Bangkok, Thailand.

*Corresponding author. E-mail: Stefan.Kappe@seattlechildrens.org (S.H.I. K).

Running Title: P36 and P52 act together

Text S1. Supplementary Methods.

Table S1. Oligonucleotides used for the creation of the *P. yoelii* transgenic parasite lines.

Table S2. The *in vivo* phenotype of Δ*P52* and Δ*P36* parasites.

Table S3. **Mass spectrometry results for the P36-P52 immunoprecipitation experiments (separate file).**

Table S4. **Antibodies used in this work.**

Figure S1. **Schematic of the cloning strategy used for the transgenic parasite lines used in this study.**

Figure S2. Both P36 and P52 are Required for the Formation of the Parasitophorous Vacuole.

Figure S3. P36^{mCherry} sporozoites have a normal gliding phenotype.

Figure S4. P36^{mCherry} sporozoites are capable of invading hepatoma cells.

Figure S5. P36 and P52 are Localized in the Sporozoite Micronemes.

Figure S6. Analysis of the co-localization of P36, P52 and TRAP.

Figure S7. P52 Co-precipitates P36.

Figure S8. P36 and P52 accumulate at the apical end upon sporozoite activation.

Text S1. Supplementary Methods

Creation of *P. yoelii* P52^{myc} –To epitope tag P52, a quadruple (4x) myc tag sequence was introduced into the b3D.DT^H.^D vector (Catalog # MRA-80 in the MR4 Malaria Research and Reference Reagent Resource Center; https://www.beiresources.org/About/MR4.aspx). *P. yoelii P52* (PlasmoDB identifier PY17X_1003600), including approximately 1.4 kb of sequence upstream of the start codon and excluding the GPI-anchor sequence, was amplified from *Py* 17XNL genomic DNA and cloned in frame upstream of the 4x myc tag. The GPI-anchor sequence and approximately 0.5 kb of downstream sequence was inserted after the 4x myc tag (Fig S1c). Approximately one kb of the 3′ untranslated region of the *P. berghei DHFR/TS* gene was added to the C-terminus of the downstream sequence to ensure stability of the recombinant messenger RNA. The resulting plasmid was linearized with *BsaI* for integration into *Py* 17XNL blood stage schizonts using standard procedures. Integration of the plasmid to create P52^{myc} gave rise to a parasite line that expressed two copies of P52, both with the endogenous promoter and one containing the 4x myc epitope tag.

Creation of α-P52 (mAbs 13G10 and 2D5) and α-P36 rabbit serum

Expression of recombinant PyP52 and PyP36 (rPyP52, rPyP36): Fragments encoding PyP52 (1302 bp, excluding N-terminal signal peptide and C-terminal GPI-anchor domain) and PyP36 (693 bp, excluding N-terminal signal peptide) were amplified from *P. yoelii* 17XNL genomic DNA by PCR. Gene-specific primer sets (PyP52 forward, 5'-gagagagactcgagaatgtgccaaatcctaatacgacca-3' and PyP52 reverse, 5'-gagagagaggaatcctcaggtaccaaacatattttgccaaaatg-3'; PyP36 forward, 5'-gggcggatatctcgagacaataaaattgctttgccctattgtc-3' and PyP36 reverse, 5'-gcggtacccgggatccttatattaaaatgtccctatctac-3') were designed to amplify the target DNA fragments, which were cloned into either pEU-E01-HisGST(TEV)-MCS-N2 vector or pEU-E01-GST-TEV-MCS-N2 vector (CellFree Sciences Co. Ltd.) as previously described (1). Plasmids were then utilized for protein production in a wheat germ cell-free protein expression system using the bilayer translation reaction method (2,3). GST-fused rPyP52 was bound to a glutathione Sepharose 4B column (GE Healthcare), followed by cleavage with a AcTEV protease (Thermo Fisher Scientific) to elute the rPyP52 protein from the column. GST-fused rPyP36 was bound to a glutathione Sepharose 4B column and eluted with elution buffer (40mM reduced glutathione, 50mM Tris-HCl, 300mM NaCl, 200mM Imidazole, 2% glycerol and pH 8.0).

α-P52 monoclonal antibody production: Purified rPyP52 was injected intraperitoneally into an 8-week old female BALB/c mouse, together with Freund's complete adjuvant on the first occasion and with Freund's incomplete adjuvant 3 weeks and 6 weeks later. Seven weeks after the initial injection, an intravenous boost with the same amount of rPyP52 in PBS (pH 7.4) was administered, and the spleen was used to produce hybridoma cell lines. After an initial screening by ELISA using rPyP52 as an antigen and immunofluorescence microscopy using acetone-fixed *P. yoelii* 17XNL sporozoites, hybridoma cell lines were cloned by two rounds of limiting dilution. Cloned cell lines (2D5, 13G10) were expanded to collect ascites in mice primed with pristane (Wako), and immunoglobulins were purified from ascitic fluid using an Ampure PA kit (GE Healthcare).

α-P36 rabbit antiserum: To generate antiserum against PyP36 with an N-terminal GST-tag, a Japanese white rabbit was immunized subcutaneously with purified rPyP36 with Freund's complete adjuvant, followed by two immunizations using rPyP36 with Freund's incomplete

adjuvant. Immunization was done at 3-week intervals and antisera were collected 14 days after the last immunization (Kitayama labes Co. Ltd.).

Creation of α-PyP36.1 and α-PyTRAP.2 monoclonal antibodies

P. yoelii P36 (PlasmoDB:PYYM_1003500) and thrombospondin-related anonymous protein (TRAP) (PlasmoDB:PYYM_1351500) were produced in suspension HEK293 cells as previously described (4). In each case, the final construct contained a tPA signal peptide (5), codon-optimized sequences encoding the extracellular domain of P36 (residues 71–356) or that of TRAP (residues 23–752), and a C-terminal His8 tag.

Immunization of Balb/cj mice, B-cell isolation, cloning of monoclonal antibodies (mAbs), and recombinant mAb production was carried out, as previously described (6). For production of recombinant mAbs encoded by Py P52- 13G10 hybridomas, hybridoma-derived RNA was isolated and used following the same cloning/production protocol (6).

Table S1. Oligonucleotides used for the creation of the *P. yoelii* transgenic parasite lines.

Oligonucleotide Sequence 5’-3’

Single and double ΔP52 and ΔP36 parasites

P52 KO 5UTR F	atgcccactattcgcctgcaggCAAATTCGTGCATGTATACAAG
P52 KO 5UTR R	gcccgcggCAAACGGTAATAGTGGACATCA
P52 KO cORF F	gcccgcggGTTGTGCATGCAAAAGAGATG
P52 KO 3UTR R	catgcacgaatttgcctgcaggCGAATAGTGGGCATATATTGAC
P52 5UTR Test 1 F	GATTGTGGAATTTCCATGTT
P52 3UTR Test 2 R	GTGTTAGGGCAAAACAGATAGA
P36 KO 5UTR F	tccatatgatcgcctgcaggGAACACATTGTTTTATTTAATCCAT
P36 KO nORF R	gcccgcggTTGGATAGTCTCTTAAGTTACATATGA
P36 KO cORF F	gcccgcggTGAAATAGATGCATATCCTGGG
P36 KO 3UTR R	aacaatgtgttccctgcaggCGATCATATGGAATAATTAAACAA
P36 5UTR Test 1 F	GCGGAGAAAAGACCATTTAAT
P36 3UTR Test 2 R	GATCAGAACGAATAACCTTTACATTGA
Casst test1 R	GCAAGGCGATTAAGTTGGGT
Casst test2 F	GGCTACGTCCCGCACGGACGAATCCAGATGG

P36^{mCherry} parasites

P36 tag 3UTR F	ATCCGCGGTATGTGATGTATGTAGAAGAGTG
P36 tag 3UTR R	TATAAAATAACAAAGAAGGGCCCAATTTACTACTTAAAATTGGAAACC
P36 tag ORF F	ATTTTAAGTAGTAAATTGGGCCCTTCTTTGTTATTTTATATGTGTATATGC
P36 tag ORF R	ATGCGGCCGCCACTTGCTTTTGTGGAGAAAATGCG
P36 Test F	GTCATATTAACACATATTTTTCCG
pDEF Test R	CCTTCTATTTCAAATTCATGTCC
pDEF Test F	CAATGATTCATAAATAGTTGGACTTG
P36 Test R	TTAAGTATTGAGGCACACCGG

P52^{Myc} parasites

P52myc 5UTR F	attaccgcggGTAGTCCGATAAAATACATCTCTAG
P52myc nORF R	attagcggccgctAAACATATTTTGCCAAAATG
P52myc cORF F	attaactagTGGTACCTCTTCTTCTTATTATGAGGTC
P52myc 3UTR R	attagaattcTCATTGCTAGTTGGTTATATA

Table S2. The *in vivo* phenotype of Δ*P52* and Δ*P36* parasites is almost as severe as the phenotype observed with the Δ*P52*Δ*P36* parasites. Summary of three independent experiments where BALB/cJ and BALB/cByJ mice were injected (i.v.) with 1 x 10^4 or 1 x10^5 sporozoites. All mice injected with wild-type sporozoites were positive for a blood infection becoming patent at day 2.5 or 3 as expected. Most of the mice injected with Δ*P36*, Δ*P52* or Δ*P52*Δ*P36* sporozoites did not become patent. However, breakthroughs for Δ*P36* and Δ*P52*Δ*P36* were observed with extended delays for the double deletion mutant.

P. yoelii genotype	No. of injected sporozoites	**BALB/cJ**		**BALB/cByJ**	
		No. of infected mice	Day of patency	No. of infected mice	Day of patency
Δp52	1 × 10^4	0/5	-	0/5	-
Δp36	1 × 10^4	0/5	-	1/5	4
Δp52Δp36	1 × 10^4	0/5	-	0/5	-
Wild type	1 × 10^4	5/5	3	5/5	3
Δp52	1 × 10^5	0/5	-	0/2	-
Δp36	1 × 10^5	1/5	4	0/2	-
Δp52Δp36	1 × 10^5	0/5	-	1/5	5
Wild type	1 × 10^5	5/5	2.5	5/5	2.5
Δp52Δp36	1 × 10^5	-	-	1/25	5

Table S4. Antibodies used in this work.

Target	Name	Type	Species	Figure #	Concentration (mg/ml)	Dilution	Source
**Primary Antibodies**							
UIS4	α-UIS4	sera	rabbit	1	-	1:250	(7)
mCherry	16D7	mAb	rat	2B	2	1:1000	Molecular Probes (Thermo)
mCherry	16D7	mAb	rat	3A, 5A, S8	2	1:500	Molecular Probes (Thermo)
mCherry	NBP2-43720	poly	rabbit	4A, 4B, 5B	0.36	1:1000	Novusbio
P52	13G10	mAb	mouse	2B, 4A, 4B, 5B	0.65	1:1000	This work
P52	13G10	mAb	mouse	3A, S5, S8	1	1:100	This work
P52	2D5	mAb	mouse	3B single	3.54	1:500	This work
P52	2D5	mAb	mouse	3B double	3.54	1:2500	This work
MTIP	α-MTIP	sera	rabbit	2B	-	1:2000	(8)
MTIP	α-MTIP	sera	rabbit	3A, 5A, S5	-	1:1000	(8)
P36	α-PyP36.1	mAb	mouse	3A, S5, S8	1	1:100	This work
P36	α-PyP36.1	mAb	mouse	3B	1.2	1:20	This work
P36	α-P36	sera	rabbit	S5	-	1:400	This work
P36	α-P36	sera	rabbit	3B	-	1:250	This work
TRAP	α-PyTRAP.2	mAb	mouse	3A, 5A, S8	1	1:100	This work
TRAP	α-PyTRAP.2	mAb	mouse	3B	1.7	1:25	This work
TRAP	α-TRAP (antirepeats)	sera	rabbit	S5	-	1:500	(9)
TRAP	α-TRAP (antirepeats)	sera	rabbit	3B	-	1:1000	(9)
TRAP	α-TRAP (antirepeats)	sera	rabbit	5B	-	1:3000	(9)
Myc Tag	9E10	mAb	mouse	S7	3.03	1:500	BioXCell
CSP	2F6-AF^{594}	mAb	mouse	S3	1	1:500	(labeled in house) (10)
CSP	2F6-AF^{647}	mAb	mouse	S4	1	1:500	(labeled in house) (10)
**Secondary Antibodies**							
Rat	α-rat HRP	poly	donkey	2B	1	1:10,000	Novex
Mouse	α-mouse HRP	poly	goat	2B, 4A, 4B, 5B, S7	0.8	1:10,000	Thermo Fisher Scientific
Rabbit	α-rabbit HRP	poly	goat	2B, 4A, 4B, 5B	0.5	1:40,000	Pierce
Rat	α-rat AF^{594}	poly	goat	3A, 5A, S8	2	1:500	Invitrogen/Thermo
Mouse	α-mouse AF^{488}	poly	goat	3A, 5A, S5, S8	2	1:500	Invitrogen/Thermo
Rabbit	α-rabbit AF^{488}	Poly	goat	1, 3A, 5A,	2	1:500	Invitrogen/Thermo
Rabbit	α-rabbit AF^{594}	poly	goat	S5	2	1:500	Invitrogen/Thermo
Mouse	α-mouse 15nm-Gold	poly	goat	3B	-	1:20	BBI Solutions
Rabbit	α-rabbit 10nm-Gold	poly	goat	3B	2	1:20	Invitrogen/Thermo

Figure S1. Schematic of the cloning strategy used for the transgenic parasite lines used in this study.

a. **Δ*P36*, Δ*P52* and Δ*P52*Δ*P36***

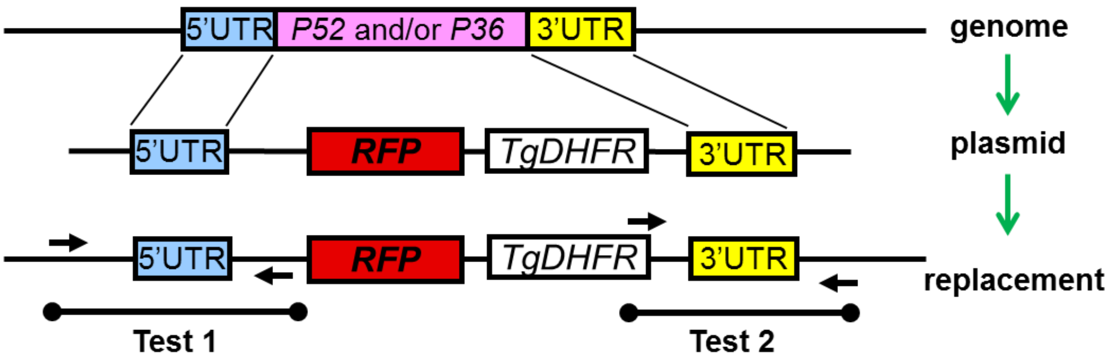

b. **P36**$^{\mathbf{mCherry}}$

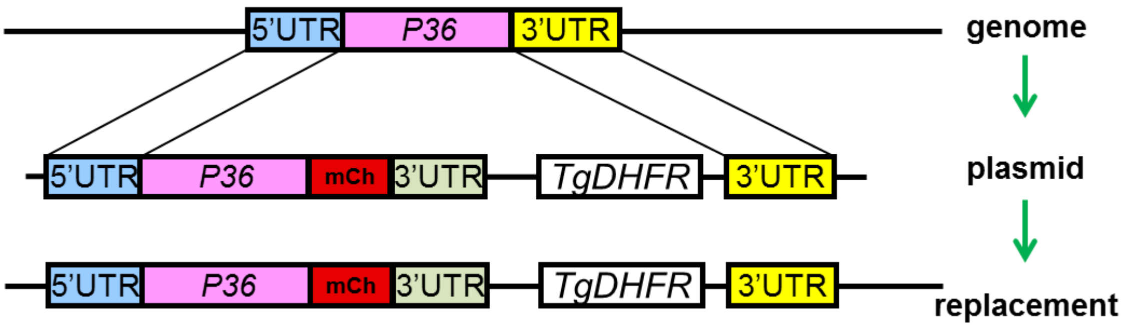

c. P52^{myc}

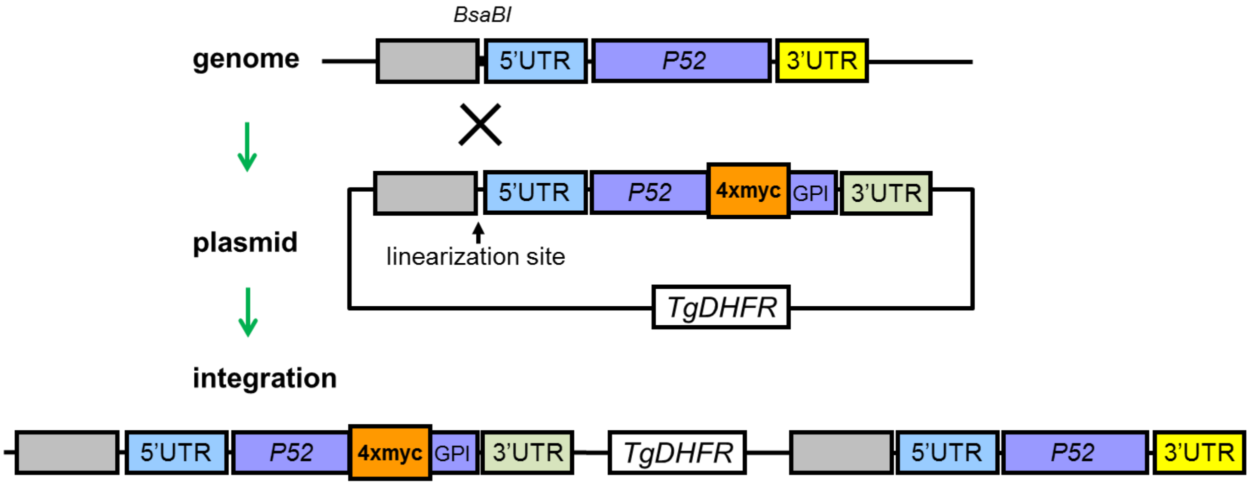

BsaBI
genome
5'UTR
P52
3'UTR
plasmid
5'UTR
P52
4xmyc
GPI
3'UTR
linearization site
TgDHFR
integration
5'UTR
P52
4xmyc
GPI
3'UTR
TgDHFR
5'UTR
P52
3'UTR

Figure S2. Both P36 and P52 are Required for the Formation of the Parasitophorous Vacuole. 3D visualization analysis of live fluorescence images (Fig. 1, right panel) by Imaris demonstrates that while all sporozoites are intracellular, only the wild-type parasite is enclosed by a filipin-labeled membrane indicating the presence of a PVM.

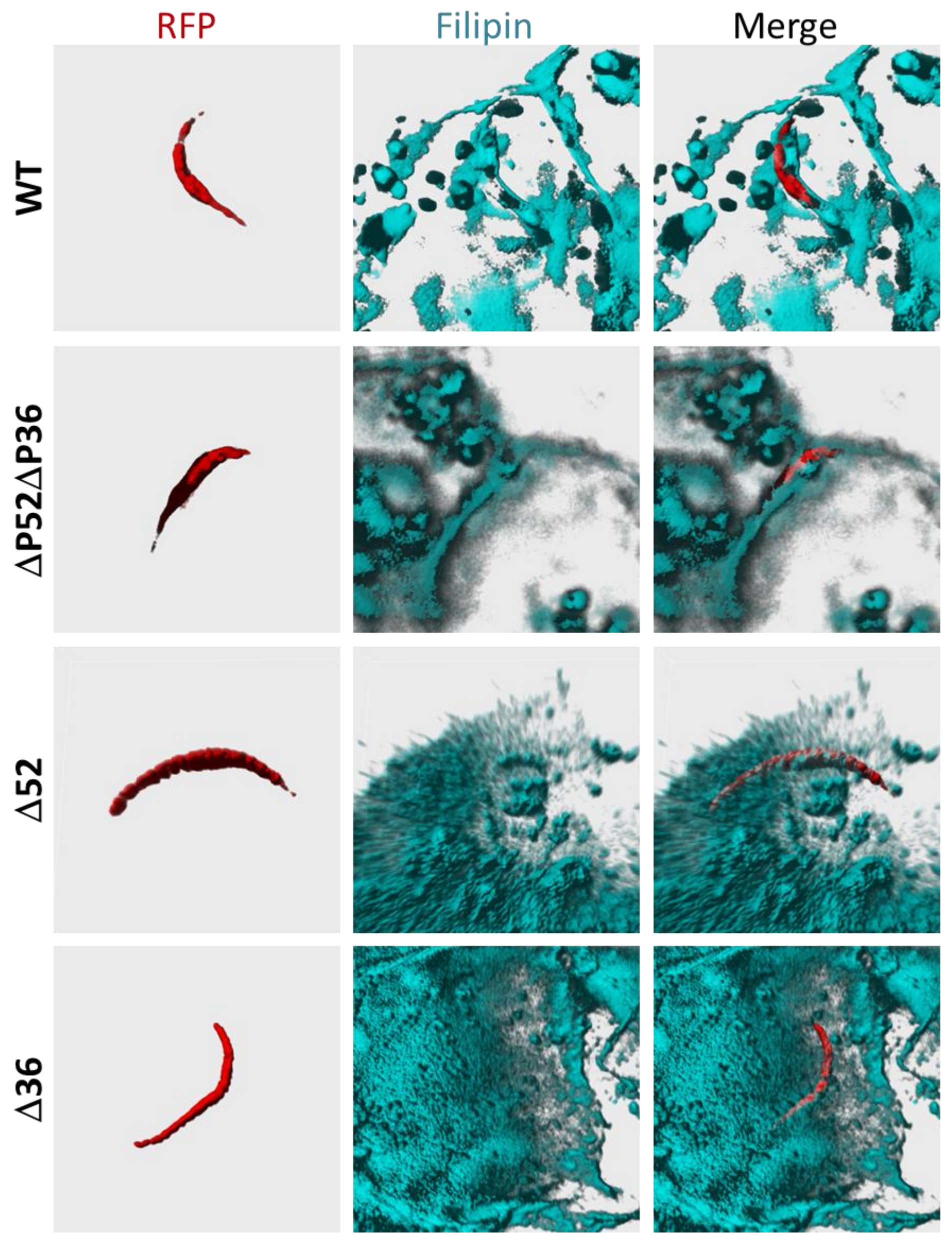

Figure S3. P36^{mCherry} sporozoites have a normal gliding phenotype. The well of a chamber slide was coated with 10 µg/ml of α-PyCSP mAb in PBS overnight at room temperature. After removal of excess antibody, the slide was placed in a wet chamber and 5 x10^5 P36^{mCherry} sporozoites activated in DMEM containing 20% FBS were placed in the well. Following incubation for 2:30 h at 37°C, the sporozoites were fixed with 4% PFA at room temperature for 15 min and washed three times with PBS. Following blocking with 1X PBS with 3% BSA for 30 min, the sample was incubated with fluorescently conjugated α-PyCSP-AF^{594} in blocking Buffer for 1 h. After washing three times with 1X PBS, the well was detached, and the slide was mounted with Vectashield HardSet. Slides were analyzed for fluorescence with a Nikon Eclipse E600 Fluorescence Microscope. (Sporozoite not shown in the left image; red arrow points at sporozoite in right image)

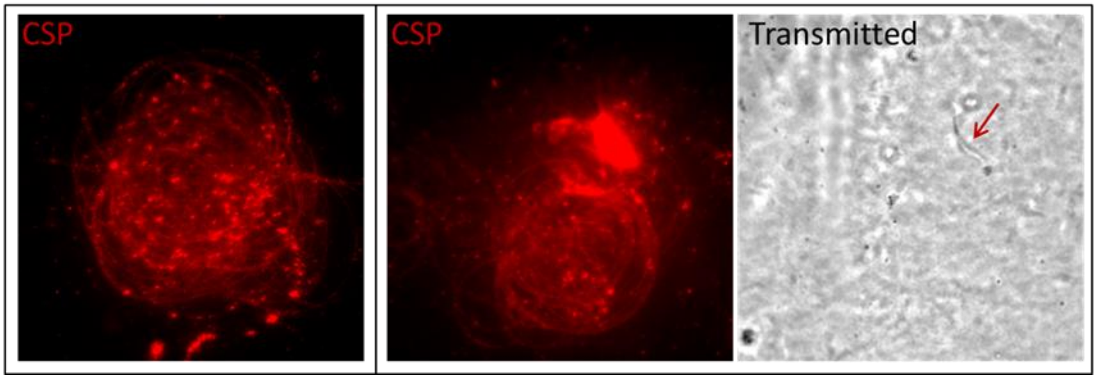

Figure S4. P36^{mCherry} sporozoites are capable of invading hepatoma cells. Analysis of sporozoite invasion *in vitro* was performed as previously described (11). Briefly, Hepa 1-6 cells were maintained in complete DMEM media (Thermo Fisher Scientific) containing 10% FBS (Sigma-Aldrich), 200 U/mL penicillin with 200 µg/mL streptomycin (Thermo Fisher Scientific), and 2.5 µg/mL Fungizone (GE Life Sciences). The day before the invasion assay, 1 x 10^5 cells were plated in each well of a 96-well plate and cells were allowed to adhere overnight. The next day 5 x 10^4 sporozoites were added to five replicate wells in complete media, incubated for 90 minutes, washed to remove non-invaded sporozoites and fixed with Perm/Fix buffer (BD Biosciences). Cells were then blocked with Perm/Wash buffer (BD Biosciences) and stained with a monoclonal antibody specific for *P. yoelii* CSP (clone 2F6) conjugated to AlexaFluor 647 (Invitrogen/Thermo). Intracellular sporozoites were detected using a BD-LSRII flow cytometer (BD Biosciences) and the percent CSP+ invaded cells expressed as a percent of that seen with wild-type sporozoites.

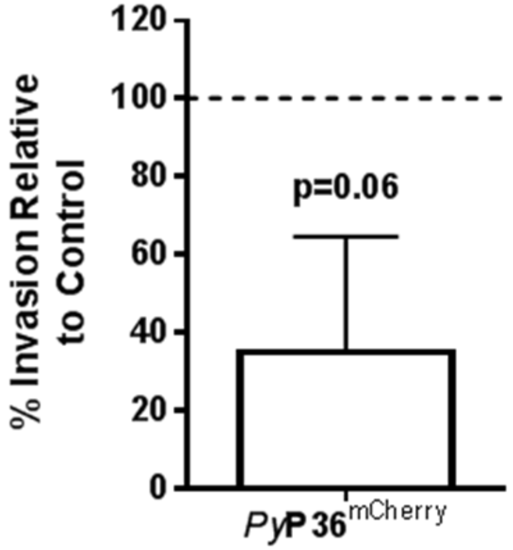

Figure S5. P36 and P52 are Localized in the Sporozoite Micronemes. Representative immunofluorescence microscopy images of **wild-type** salivary gland sporozoites show the internal localization of P36 compared to the micronemal proteins P52 and TRAP and in contrast to the inner membrane complex protein MTIP. Nuclei were stained with DAPI. Primary antibodies used: rabbit α-MTIP, mouse α-PyP36.1, mouse α-PyP52 (13G10), rabbit α-TRAP and rabbit α-PyP36. Secondary fluorescently-labeled antibodies: α-rabbit-AF^{594}and α-mouse-AF^{488} (Scale bar: 5 um)

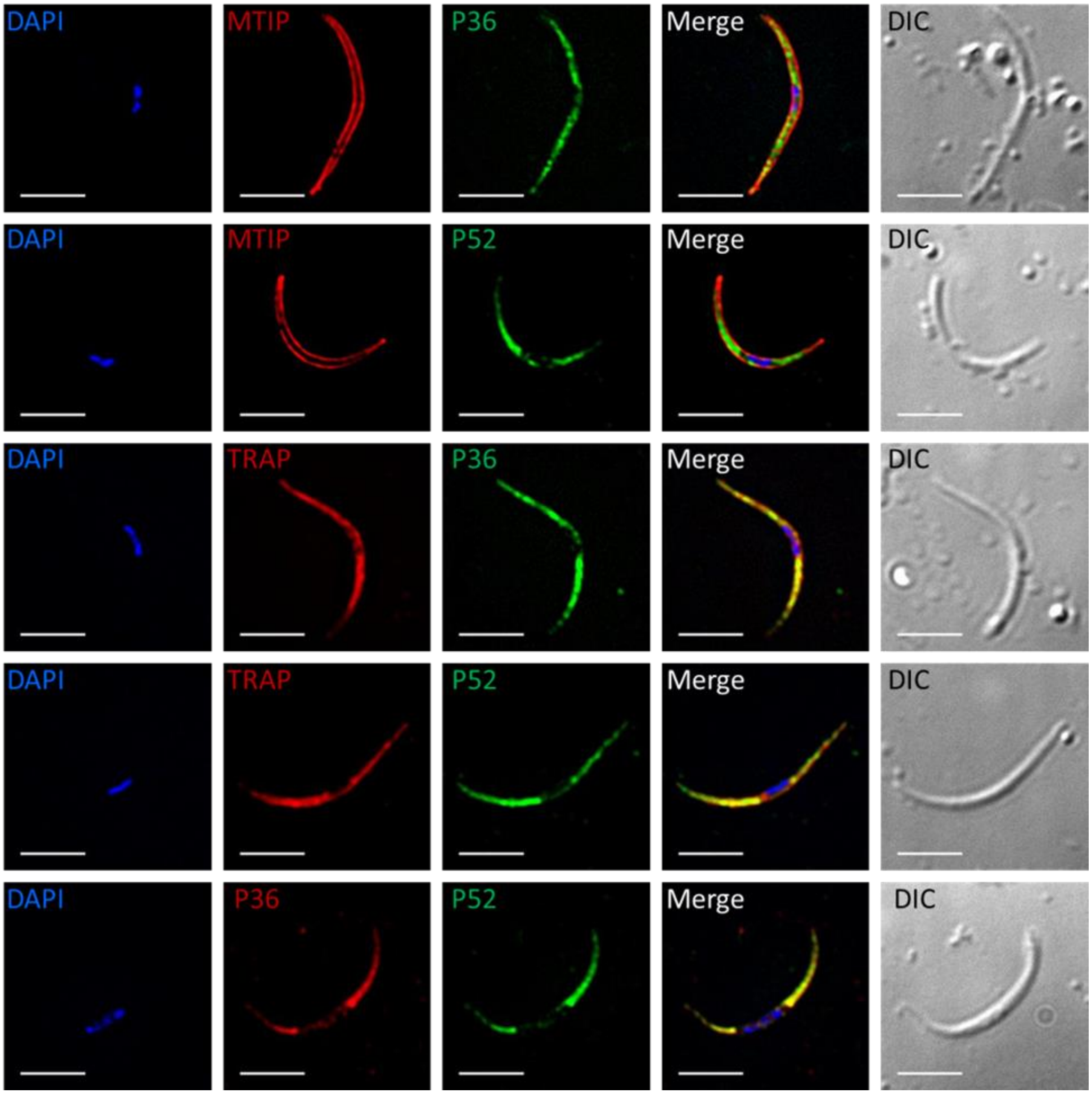

Figure S6. Analysis of the co-localization of P36, P52 and TRAP observed in electron microscopy images of labeled *P. yoelii* P36^{mCherry} sporozoite slices (Figure 3B). The table shows the number of micronemes counted with no labeling (blank), and single or dual antibody labeling in 10 (P36 and P52; P36 and TRAP) and 7 (P52 and TRAP) sporozoite slices. The average number of micronemes per sporozoite slice counted within each category is also shown with its corresponding standard deviation. The last column shows the percentage of the total microneme count that is unlabeled, single-labeled or double-labeled in each antibody combination. Micronemes were initially physically delimited based on their discrete shape regardless of labeling; all discrete micronemes were taken into account and ultimately classified according to label. The calculations of the frequency of a given protein found co-localized within the sporozoite are shown. The data from the table is graphically displayed in the chart shown in Fig. 3B.

	Labeled with	# of Micronemes Counted	Average # per spz	Std Dev	%
	Blank	57	5.7	2.8	18
	P36 and P52	103	10.3	2.5	33
**P36 and P52**	P36	107	10.7	3.6	34
	P52	48	4.8	2.0	15
	Total Counted	315	31.5	5.9	100
	Blank	71	7.1	2.7	20
	P36 and TRAP	91	9.1	4.0	26
**P36 and TRAP**	P36	65	6.5	3.2	18
	TRAP	128	12.8	4.1	36
	Total Counted	355	35.5	10.1	100
	Blank	12	1.7	1.3	5
	P52 and TRAP	119	17.0	8.2	46
**P52 and TRAP**	P52	17	2.4	1.1	7
	TRAP	110	15.7	2.6	43
	Total Counted	258	36.9	8.7	100

Frequency of dual labeling (F):

$$\text{P52 with P36} = \frac{\text{(P36 and P52)}}{[\text{P52} + \text{(P36 and P52)}]} = \frac{103}{[48 + 103]} = 0.68$$

$$\text{P36 with P52} = \frac{\text{(P36 and P52)}}{[\text{P36} + \text{(P36 and P52)}]} = \frac{103}{[107 + 103]} = 0.49$$

$$\text{P36 with TRAP} = \frac{\text{(P36 and TRAP)}}{[\text{P36} + \text{(P36 and TRAP)}]} = \frac{91}{[65 + 91]} = 0.58$$

$$\text{P52 with TRAP} = \frac{\text{(P52 and TRAP)}}{[\text{P52} + \text{(P52 and TRAP)}]} = \frac{119}{[17 + 119]} = 0.875$$

Figure S7. P52 Co-precipitates P36. The western blot analysis shows the IP of P52^{Myc} from lysates of *P. yoelii* sporozoites (IN) genetically modified to express a quadruple myc tag upstream of the GPI-anchor of P52 as described. *P. yoelii* GFP-Luc (12) sporozoite lysate was used as wild-type control for unspecific binding in this experiment (left panel). The sample preparation, IP and blotting were done as described but using Myc-Trap®-MA (Chromotek) and probing with mouse α-Myc and α-mouse-HRP antibodies. Mass spectrometry analysis of immunoprecipitated P52^{Myc} showed that P36 peptides were co-precipitated with P52 peptides as summarized in the table below. (See Table S3 for a complete list of results).

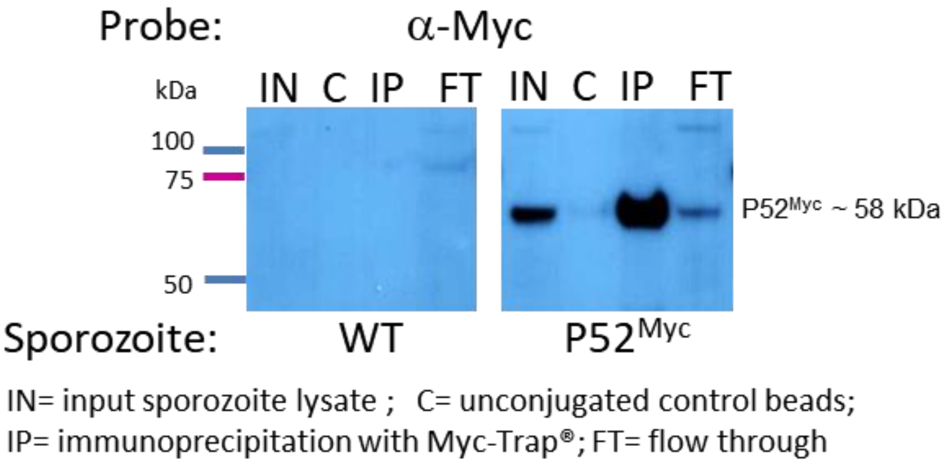

Gene ID	Protein	Description	P52^{Myc} sporozoites		WT (GFP-Luc) sporozoites	
			PSMs	Peptides	PSMs	Peptides
PY17X_1003600	P36p	6-cysteine protein, putative (P52)	63	15	0	0
PY17X_1003500	P36	6-cysteine protein	11	6	0	0
PY17X_0822200	HSP70-2	heat shock protein 70, putative	78	25	3	3

The total number of peptide spectrum matches (PSMs) and unique peptides identifying the proteins in each experiment is given. PSMs and peptides were only counted for peptides that were not shared with non-*Plasmodium* proteins, e.g. mosquito.

Figure S8. P36 and P52 accumulate at the apical end upon sporozoite activation. **(A)** 3D volume visualization of P36^{mCherry} and TRAP labeling observed in the sporozoite shown in Fig. 5A last row. **(B)** Representative immunofluorescence microscopy images of activated sporozoites within the context of a Hepa1-6 cell invasion assay (90 min incubation) showing the accumulation of P36^{mCherry} and P52 at the apical end of the sporozoite. Nuclei were stained with DAPI. Primary antibodies used: rat α-mCherry, mouse α-PyP36.1 and mouse α-P52 (13G10). Secondary fluorescently-labeled antibodies: α-rat-AF^{594} and α-mouse-AF^{488} (Scale bar: 5 um). 3D volume visualization was done with Imaris.

(A)

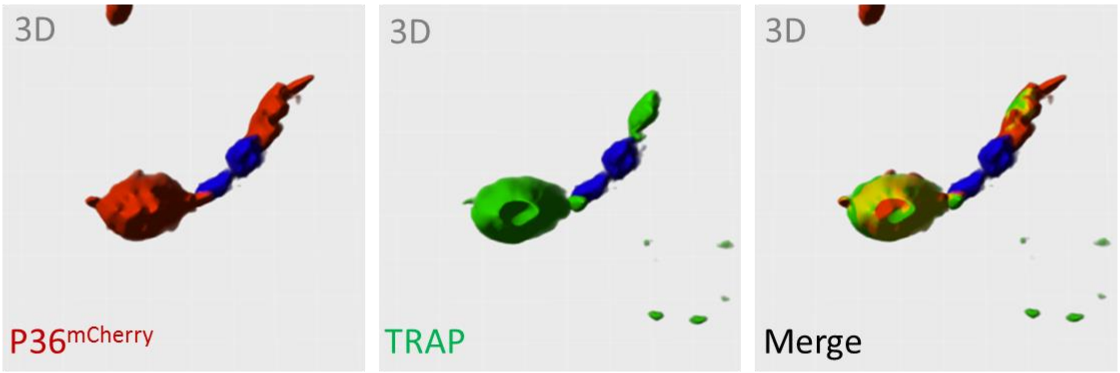

(B)

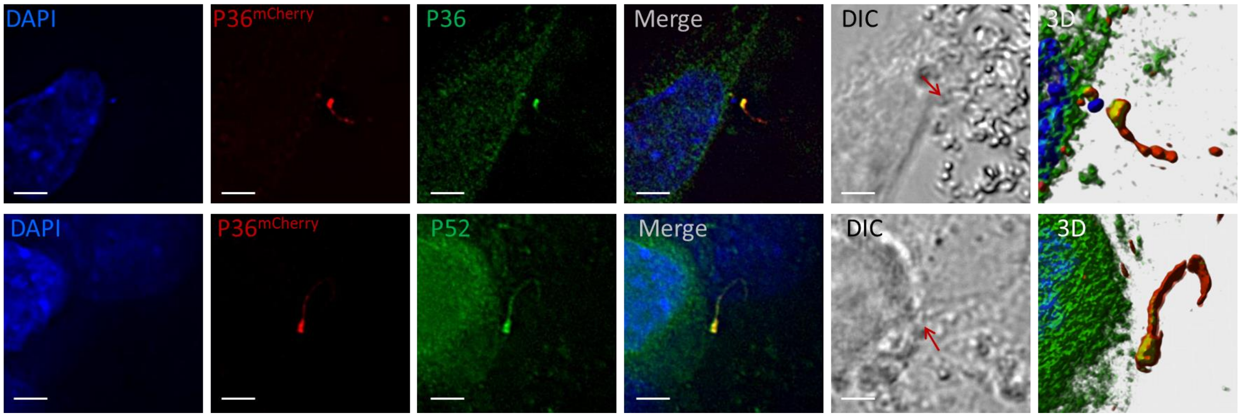

References

1. Tsuboi, T., Takeo, S., Iriko, H., Jin, L., Tsuchimochi, M., Matsuda, S., Han, E. T., Otsuki, H., Kaneko, O., Sattabongkot, J., Udomsangpetch, R., Sawasaki, T., Torii, M., and Endo, Y. (2008) Wheat germ cell-free system-based production of malaria proteins for discovery of novel vaccine candidates. *Infect Immun* **76**, 1702-1708
2. Sawasaki, T., Ogasawara, T., Morishita, R., and Endo, Y. (2002) A cell-free protein synthesis system for high-throughput proteomics. *Proc Natl Acad Sci U S A* **99**, 14652-14657
3. Takai, K., Sawasaki, T., and Endo, Y. (2010) Practical cell-free protein synthesis system using purified wheat embryos. *Nat Protoc* **5**, 227-238
4. Kaushansky, A., Douglass, A. N., Arang, N., Vigdorovich, V., Dambrauskas, N., Kain, H. S., Austin, L. S., Sather, D. N., and Kappe, S. H. (2015) Malaria parasites target the hepatocyte receptor EphA2 for successful host infection. *Science* **350**, 1089-1092
5. Wang, J. Y., Song, W. T., Li, Y., Chen, W. J., Yang, D., Zhong, G. C., Zhou, H. Z., Ren, C. Y., Yu, H. T., and Ling, H. (2011) Improved expression of secretory and trimeric proteins in mammalian cells via the introduction of a new trimer motif and a mutant of the tPA signal sequence. *Appl Microbiol Biotechnol* **91**, 731-740
6. Carbonetti, S., Oliver, B. G., Vigdorovich, V., Dambrauskas, N., Sack, B., Bergl, E., Kappe, S. H. I., and Sather, D. N. (2017) A method for the isolation and characterization of functional murine monoclonal antibodies by single B cell cloning. *J Immunol Methods*
7. Kaiser, K., Matuschewski, K., Camargo, N., Ross, J., and Kappe, S. H. (2004) Differential transcriptome profiling identifies Plasmodium genes encoding pre-erythrocytic stage-specific proteins. *Molecular microbiology* **51**, 1221-1232
8. Bergman, L. W., Kaiser, K., Fujioka, H., Coppens, I., Daly, T. M., Fox, S., Matuschewski, K., Nussenzweig, V., and Kappe, S. H. (2003) Myosin A tail domain interacting protein (MTIP) localizes to the inner membrane complex of Plasmodium sporozoites. *J Cell Sci* **116**, 39-49
9. Sultan, A. A., Thathy, V., Frevert, U., Robson, K. J., Crisanti, A., Nussenzweig, V., Nussenzweig, R. S., and Menard, R. (1997) TRAP is necessary for gliding motility and infectivity of plasmodium sporozoites. *Cell* **90**, 511-522
10. Sinnis, P., De La Vega, P., Coppi, A., Krzych, U., and Mota, M. M. (2013) Quantification of sporozoite invasion, migration, and development by microscopy and flow cytometry. *Methods Mol Biol* **923**, 385-400
11. Kaushansky, A., Rezakhani, N., Mann, H., and Kappe, S. H. (2012) Development of a quantitative flow cytometry-based assay to assess infection by Plasmodium falciparum sporozoites. *Mol Biochem Parasitol* **183**, 100-103
12. Miller, J. L., Murray, S., Vaughan, A. M., Harupa, A., Sack, B., Baldwin, M., Crispe, I. N., and Kappe, S. H. (2013) Quantitative bioluminescent imaging of pre-erythrocytic malaria parasite infection using luciferase-expressing Plasmodium yoelii. *PLoS One* **8**, e60820
